# Effectiveness of Mental Health and Wellbeing Interventions for Children and Young People in Foster, Kinship, and Residential Care: Systematic Review and Meta-Analysis

**DOI:** 10.1177/15248380241227987

**Published:** 2024-02-16

**Authors:** Rob Trubey, Rhiannon Evans, Sarah McDonald, Jane Noyes, Mike Robling, Simone Willis, Maria Boffey, Charlotte Wooders, Soo Vinnicombe, G. J. Melendez-Torres

**Affiliations:** 1 Cardiff University, UK; 2 Bangor University, UK; 3 The Fostering Network in Wales, London, UK; 4 University of Exeter, UK

**Keywords:** effectiveness, outcome, intervention, mental health, foster care, residential care, systematic review

## Abstract

The mental health and wellbeing of children and young people who have been in care, primarily foster care, kinship care or residential care, remains a public health priority. The Care-experienced cHildren and young people’s Interventions to improve Mental health and wEll-being outcomes Systematic review (CHIMES) synthesized evidence for the effectiveness of interventions targeting: subjective wellbeing; mental, behavioral and neurodevelopmental disorders; and suicide-related outcomes. Searches were conducted in 16 bibliographic databases and 22 websites between 1990 and 2022. This was supplemented by citation tracking, screening of relevant systematic reviews, and expert recommendation. We identified 35 interventions, with 44 evaluations via randomized controlled trials. Through meta-analyses, we found that interventions have a small beneficial impact on a variety of mental health outcomes in the short term (0–6 months). Interventions improved total social, emotional, and behavioral problems (*d* = −0.15, 95% CI [−0.28, −0.02]), social-emotional functioning difficulties (*d* = −0.18, 95% CI [−0.31, −0.05]), externalizing problem behaviors (*d* = −0.30, 95% CI [−0.53, −0.08]), internalizing problem behaviors (*d* = −0.35, 95% CI [−0.61, −0.08]); and depression and anxiety (*d* = −0.26, 95% CI [−0.40, −0.13]). Interventions did not demonstrate any effectiveness for outcomes assessed in the longer term (>6 months). Certainty of effectiveness was limited by risk of bias and imprecision. There was limited available evidence for interventions targeting subjective wellbeing and suicide-related outcomes. Future intervention design and delivery must ensure that programs are sufficient to activate causal mechanisms and facilitate change. Evaluation research should use a robust methodology.

PROSPERO Registration: CRD42020177478

## Introduction

Children and young people with experience of living in care are a diverse population. They can include individuals currently in statutory care or with a history of care placement. Placement types can comprise foster care, kinship care, residential care or other living situations where parental responsibility is transferred to another adult (e.g., Special Guardianship Orders) (Allik et al., 2021). The mental health and wellbeing of this population remains a health and social care priority. They have a higher rate of diagnosable mental health problems when compared to non-care-experienced samples (Dubois-Comtois et al., 2021; Engler et al., 2022; Seker et al., 2021), and are at increased risk of poor subjective wellbeing (Long et al., 2017) and suicide attempts (Evans et al., 2017). While there is limited longitudinal research, recent evidence from the UK reports that individuals who have been in care have excess mortality in adulthood up to 42 years later, which is attributable to a higher level of self-harm, accidents, and other mental health and behavioral factors (Murray et al., 2020).

In recent years, there has been a significant increase in interventions targeting the mental health and wellbeing of care-experienced populations. Primarily originating in the USA, the majority of programs have focused on intrapersonal and interpersonal mechanisms of change, notably targeting parenting knowledge, competency, and self-efficacy among foster and kinship carers (Chamberlain, 2003; Green et al., 2014; Price Joseph, 2009; Price Joseph et al., 2019). Other interventions have attended to the social and emotional competencies of children and young people (Taussig & Culhane, 2010; Taussig et al., 2019), while a smaller number of approaches have addressed organizational ethos within social care contexts (Izzo et al., 2016). The evidence-base for intervention remains mixed, as reported across a number of literature and systematic reviews (Bergström et al., 2019; Everson-Hock et al., 2012; Greeson et al., 2020; Hambrick et al., 2016; Kerr & Cossar, 2014; Marsh, 2017; O’Higgins et al., 2018; Solomon et al., 2017; Sullivan & Simonson, 2016). However, recent National Institute for Health and Care reviews and associated practice guidelines have indicated sufficient evidence to recommend a select range of approaches, which largely focus on building positive relationships, training carers, and generating expertise in attachment and trauma-informed approaches among practitioners (National Institute for Health and Care Excellence, 2021b).

Despite progress in intervention research, there remain a number of limitations with the evidence-base, many of which being related to extant evidence syntheses. First, many reviews have lacked a robust methodology (Luke et al., 2014), particularly in relation to meta-analysis, quality appraisal, and the generation of, for example, GRADE evidence summaries to support policy and practice recommendations. Second, syntheses tend to report on a small number of outcomes, notably specific and discrete diagnosable mental health problems (e.g., depression) (Marsh, 2017; Solomon et al., 2017). There has been limited effort to synthesize evidence for a broader range of outcomes, including subjective wellbeing and suicide.

Third, and perhaps most importantly, the effectiveness of interventions according to different follow-up measurement timepoints has not been extensively considered. Evaluation research has explored the challenges of introducing and sustaining change in complex social systems (Moore et al., 2018). While limited, syntheses of intervention process evaluations have reported a range of barriers to participant engagement and continued responsiveness, including a high number of care placement and educational transitions (National Institute for Health and Care Excellence, 2021a). Thus, it is important to understand if programs that have varying times to follow-up report different levels of effectiveness, as participants will likely be at different stages of interacting with the intervention and the wider social system in which it has been delivered.

Taking these limitations together, there is an obvious need to generate high quality and relevant evidence of effectiveness through standard systematic review methodology. Such a review would benefit from considering effects for a broad range of mental health-related outcomes and for interventions assessing outcomes at different timepoints.

### Review Aim

The Care-experienced cHildren and young people’s Interventions to improve Mental health and wEll-being outcomes Systematic review (CHIMES) was a mixed-methods review that synthesized international evidence on intervention theories, processes, outcomes, and economic effects (Evans et al., 2021). The purpose of this review and meta-analysis was to:

- Evaluate the effectiveness of interventions evaluated via randomized controlled trials (RCTs) for improving mental health and wellbeing outcomes for care-experienced children and young people

## Method

The CHIMES review methodology is described in the study protocol (Evans et al., 2021) and the PROSPERO registry of systematic reviews (CRD42020177478), and an evidence map (Evans, MacDonald et al., 2023) and qualitative review and synthesis (Macdonald et al., 2023) have been reported elsewhere. The outcome synthesis is reported in accordance with the Preferred Reporting Items for Systematic reviews and Meta-Analyses statement (Supplemental Appendix A) (Liberati et al., 2009).

### Stakeholder Consultation

Consultation was conducted with three stakeholder groups on commencement of the review: two groups of care-experienced young people; and one group of fostering team managers. These consultations refined and confirmed the scope of the review in terms of priority outcomes for synthesis, with an identified need to consider the effectiveness of interventions targeting wellbeing and suicide-related outcomes. They also explored systematic challenges to longer term delivery of interventions, and sustained improvement in outcomes, which informed the decision to conduct meta-analysis according to two time-points from baseline.

### Eligibility Criteria

The parameters of the review were specified in accordance with the Population, Intervention, Comparator, Outcome and Study Design framework. *Population*: Care-experienced children and young people (≤25 years old), or the individuals, organizations and communities that might impact them. Care was defined as foster care, formal kinship care, residential care, or other statutory transfer of parental responsibility to another adult. *Intervention*: Mono-component or multi-component, operating across one or multiple socio-ecological domains (i.e., intrapersonal, interpersonal, organizational, community, and policy). *Comparator*: Usual care or active control. *Outcomes*: (a) Subjective wellbeing (life satisfaction, quality of life, and wellbeing); (b) Mental, behavioral or neurodevelopmental disorders as specified by the International Classification of Disease (ICD)-11; and (c) suicide-related outcomes (self-harm, suicidal ideation, and suicide). *Study Design*: Outcome evaluation conducted via RCT study design with no-treatment control group. Eligibility was limited to high income countries.

### Evidence Identification

Study reports were identified from 16 electronic bibliographic databases: Applied Social Sciences Index and Abstracts; British Education Index; Child Development & Adolescent Studies; CINAHL; Embase; Education Resources Information Center; Cochrane Central Register of Controlled Trials; Cochrane Database of Systematic Reviews; Health Management Information Consortium; International Bibliography of the Social Sciences; Medline; PsycINFO; Scopus; Social Policy & Practice; Sociological Abstracts; and Web of Science. Gray literature was identified from 22 relevant health care and social care websites. Searches were undertaken from 1990 which marked the ratification of the United Nations Convention on the Rights of the Child (UNICEF, 1989), and an international expansion in social care provision for children and young people. There was no language restriction. Searches were conducted May–June 2020, and updated April–May 2022. A search strategy was developed and tested in Ovid MEDLINE before being adapted to the functionality of each database and website (Supplemental Appendix A). In addition, a total of 32 subject experts and 17 third sector organizations were contacted for recommendations. Relevant systematic reviews were searched, study reports of potential interest were screened and citation tracking was conducted with included study reports.

### Study Selection

Retrieved study reports were exported to EndNote for de-duplication and then imported to the Evidence for Policy and Practice Information and Co-ordinating (EPPI) Centre’s review software EPPI-Reviewer version 4.0 (EPPI-Centre) for management. One reviewer screened study titles for clearly irrelevant retrievals. Irrelevant reports were checked by a second reviewer. Two reviewers screened title and abstracts independently and in duplicate. The same process was followed for full texts. A screening proforma was developed and calibrated with a subset of studies, which then guided eligibility assessments.

### Data Extraction and Data Items

A standardized data extraction form was developed and calibrated in EPPI-Reviewer 4. Extraction items were converted into a coding tree that included selectable a priori defined items and free text coding. A subset (10%) of study reports were extracted independently and in duplicate, with the remainder being coded by one reviewer and checked by a second. Any discrepancies were resolved through discussion.

Evaluations were extracted according to the primary extraction domains: study design; population (setting; target population; inclusion and exclusion criteria; intervention participants; group comparability at baseline; and baseline difference); study arms and duration; analysis; effectiveness outcomes; mediators; and moderators. We also extracted secondary domains of: method of recruitment; method of randomization; unit of randomization; cluster randomization; blinding; allocation sequence; allocation concealment; total sample size; and power calculation.

### Risk of Bias Assessment

Evaluations were appraised with the Cochrane risk-of-bias tool for randomized trials (RoB 2) (Higgins et al., 2019). Assessments were undertaken independently and in duplicate by two reviewers, with disagreement resolved through discussion or recourse to a third member of the review team.

### Summary of Measures

We classified outcomes according to protocol-specified outcome domains: child subjective wellbeing; child mental, behavioral, and neurodevelopmental disorders; and child suicide-related outcomes. Within these categories, we defined several mutually exclusive sub-categories and harmonized across studies to classify study-reported effects. These are summarized in Table 1.

**Table 1.** Summary of Outcome Domains and Sub-Domains.

Outcome Domain	Sub-Domain	Description
Subjective wellbeing	Quality of life	Reports of children and young people’s quality of life, or health-related quality of life.
	Subjective wellbeing	Any subjective reports of children and young people’s personal wellbeing, excluding quality of life and subjective mental health.
	Life satisfaction	Reports of children and young people’s overall satisfaction with life, as distinct from subjective wellbeing or mental health.
Mental, behavioral, and neurodevelopmental disorders	Total social, emotional, and behavioral problems	Aggregate scores of all social, emotional and behavioral problems as measured by for example, CBCL or SDQ, or measures of total problem behaviors per day (e.g., PDR).
	Total social-emotional functioning and/or impaired functioning	Included measures of social-emotional functioning, global functioning, “total difficulties,” peer problems, conduct problems, emotional regulation and mental health functioning.
	Internalizing behavior problems	Behavior problems or disorders manifested in children’s internal psychological state. Included measures of withdrawal, somatic complaints, but excluded anxiety/depression. Typically reported as a sub-scale of total problem behaviors in, for example, CBCL.
	Externalizing behavior problems	Behavior problems or disorders manifested in children’s outward behavior. Included measures of anger, aggression, rule-breaking, “delinquency”. Typically reported as a sub-scale of total problem behaviors in, for example, CBCL.
	Anxiety and depression	Reports of anxiety or depression, as measured by DSM or reported as sub-scales of standardized measures such as CBCL.
	Stress, post-traumatic stress and trauma	Including measures of post-traumatic symptoms or severity and dissociation, measured by, for example, TSCYC.
	Attachment disorder	Diagnoses of attachment disorders including reactive attachment disorder.
	Attention and hyperactivity disorder	Measures of hyperactivity and inattention, as measured by, for example, SDQ.
Self-harm and suicide		Outcomes relating to suicidal ideation, self-harm, attempted suicide, or suicide.

*Note.* CBCL = Child Behavioral Checklist; PDR = Parent Daily Report; SDQ = Strengths and Difficulties Questionnaire; TSCYC = Trauma Symptom Checklist for Young Children.

### Outcome Synthesis

A narrative summary and descriptive tables were constructed to present the results of outcome evaluations. Meta-analyses were conducted for outcome sub-domains for which there were sufficient effect sizes available. This was for sub-domains of mental, behavioral or neurodevelopmental disorders, as specified by ICD-11. Within each sub-domain, meta analyses were conducted separately for “short-term” outcomes (outcomes measured up to and including 6 months after baseline), and “long-term” outcomes (outcomes measured more than 6 months after baseline). There was not an adequate number of studies to conduct meta-analyses for the outcome domains of subjective wellbeing or suicide-related outcomes.

For the meta-analysis, effect estimates were extracted. Estimates from cluster randomized trials were checked for unit of analysis issues.

Robust variance estimation meta-analyses were undertaken according to outcome and timepoint, considering up to 6 months from baseline as short-term outcomes, and outcomes measured beyond 6 months to be longer-term. Robust variance estimation meta-analysis is a method that permits the inclusion of more than one effect estimate per study in a meta-analysis; this contrasts with standard meta-analysis models that assume independence between individual effect estimates. It is common in meta-analysis of psychosocial interventions for outcome evaluations to present multiple relevant effect estimates per outcome (e.g., multiple estimates of child behavioral problems per respondent). This method permitted use of all relevant information from included studies. Within each meta-analysis, heterogeneity was examined with a combination of Cochran’s *Q*, tau-squared and *I*^2^. We undertook a random-effects meta-analysis due to the expected high levels of clinical heterogeneity in interventions and populations. All meta-analyses were undertaken in Stata v 17 (Statacorp, 2023) using -robumeta- (Pustejovsky & Tipton, 2022).

### GRADE Assessment

Certainty of evidence was assessed with use of GRADE tools for RCTs (Guyatt et al., 2011; Ryan & Hill, 2016). Certainty was assessed for short-term and long-term outcome sub-domains relating to: subjective wellbeing; mental, behavioral, and neurodevelopmental disorders; and suicide-related outcomes. An assessment was made to ascertain if an individual study was biased or unbiased for each outcome, which was largely derived from the quality appraisals. RCTs had a baseline rate of high certainty and non-randomized studies a low certainty rating. Certainty was rated down according to: risk of bias; imprecision; inconsistency; indirectness; and publication bias. Certainty was rated up for: large magnitude of effect; dose-response gradient; and residual confounding would decrease the magnitude of effect (where this is an effect). Certainty of the evidence per outcome was assessed according to very low, low, moderate, and high.

## Results

### Study Selection

**Figure 1.**
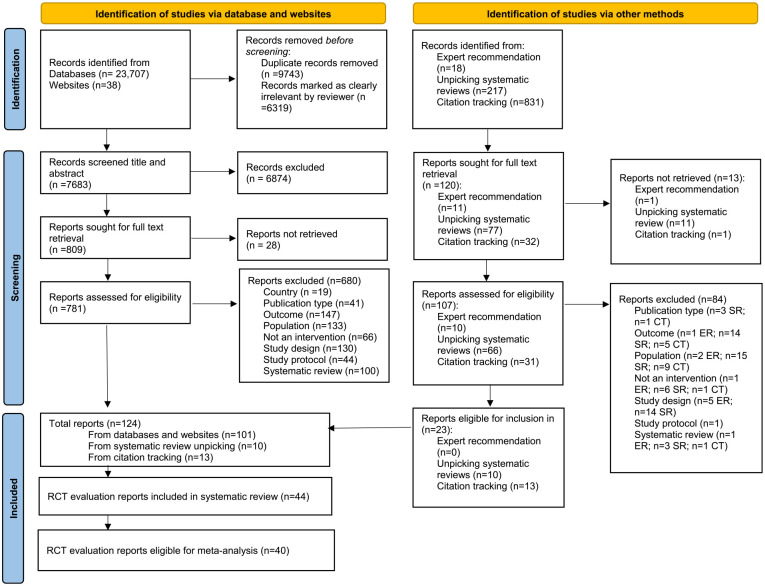
Preferred Reporting Items for Systematic reviews and Meta-Analyses flow diagram of study report retrieval.

Of 7,683 unique retrieved records, we included 44 eligible RCT evaluations of 35 interventions (Figure 1). Four evaluations were not eligible to be included in meta-analysis due to insufficient available data (Supplemental Appendix B).

### Study Characteristics

Study characteristics are summarized in Table 2 and detailed in full in Supplemental Appendix C. Of the 44 synthesized RCT evaluations, the majority (*n* = 25) were conducted in the United States, with eight evaluations conducted in the United Kingdom. Interventions primarily operated across the intrapersonal and interpersonal domains. Six interventions, with six associated study reports, targeted the intrapersonal domain (Betzalel & Shechtman, 2010; Clark Hewitt et al., 1994; Geenen et al., 2012; Jee et al., 2015; Reddy Sheethal et al., 2013; Schuurmans Angela et al., 2018). Twenty-one interventions, with 28 associated evaluations, targeted the interpersonal domain. Seven interventions, with nine associated study reports, targeted both domains (Bittman et al., 2009; Crespo et al., 2020; Dozier et al., 2006; Haight et al., 2010; Marquis, 2014; Midgley et al., 2019; Sprang, 2009; Taussig & Culhane, 2010; Taussig et al., 2019). There was one RCT evaluation of an intervention targeting the interpersonal, organizational, and community domains. The Fostering Individualized Assistance Program delivers intensive, personalized support services across agencies (Clark Hewitt et al., 1994). Only one RCT evaluation assessed a policy-level intervention: Head Start (Lipscomb et al., 2013). The duration of interventions ranged from 3 weeks to 18 months. Most interventions lasted a minimum of 2 months, with 13 interventions (and 17 associated evaluations), lasting for 5 months or more.

**Table 2.** Summary of Included Study Characteristics.

Domain	*N*	%
Country		
USA	25	56.8
UK	8	18.2
Netherlands	3	6.8
Belgium	2	4.5
Australia	2	4.5
Portugal	1	2.3
Canada	1	2.3
Germany	1	2.3
Israel	1	2.3
Socio-ecological domain targeted		
Interpersonal	28	63.6
Intrapersonal	5	11.4
Interpersonal and intrapersonal	9	20.5
Organizational and community	1	2.3
Policy	1	2.3
Primary target population		
Children and young people	15	34.1
Foster carers	9	20.5
Biological parents	1	2.3
Multiple	19	43.2
Intervention duration		
2–7 weeks	8	18.2
8–16 weeks	19	43.2
17–20 weeks	0	0.0
>20 weeks	17	38.6
Children and young people age group		
Infants/pre-school (0–5)	8	18.2
Younger children (6–11)	29	65.9
Young adolescents (12–16)	11	25.0
Older adolescents (>16)	3	6.8
Follow-up assessment period		
Short-term only (0–6 months)	26	59.1
Long-term only (>6 months)	15	34.1
Short and long-term	3	6.8

### Risk of Bias

Risk of bias assessments for the 44 included RCT studies are shown in Supplemental Appendix D. In total, six RCT evaluations were judged to have a high overall risk of bias (Alderson et al., 2020; Biehal et al., 2012; Job et al., 2022; Lipscomb et al., 2013; Mezey, Robinson et al., 2015; Suomi et al., 2020), with the remainder evaluated as having “some concern” of overall bias.

### Outcome Synthesis

#### Subjective Wellbeing

No evaluations assessed subjective wellbeing or life satisfaction. Three evaluations of two interventions assessed quality of life with child-reported measures at short and long-term follow-up (Crespo et al., 2020; Taussig & Culhane, 2010; Taussig et al., 2019). Fostering Healthy Futures (FHF) reported significantly better quality of life at post-intervention (5 months post baseline) compared to the control group (*d* = 0.42, 95% CI [0.12, 0.71]) (Taussig & Culhane, 2010). No group differences existed at 6-month follow-up in the same cohort, and there were no significant differences observed in long-term quality of life measures in a later extension of the same study (Taussig et al., 2019). No group differences in quality of life were observed in the Wave-by-Wave intervention (Crespo et al., 2020). There were insufficient data available to conduct a meta-analysis for this outcome domain.

#### Mental, Behavioral, and Neurodevelopmental Disorders

##### Total Social, Emotional and Behavioral Problems

**Figure 2.**
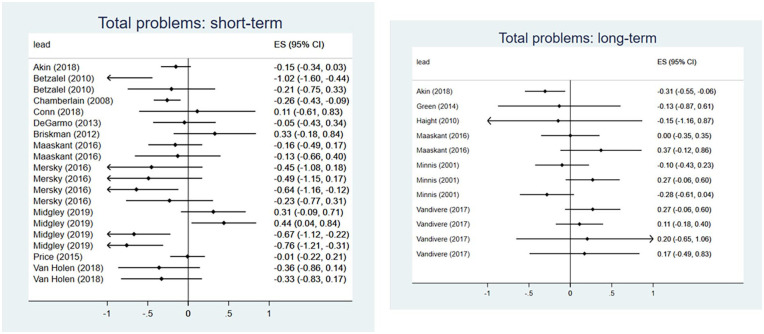
Meta-analysis forest plots for total social, emotional, and behavioral problems: short-term and long-term outcomes.

Seventeen evaluations of 14 interventions reported outcomes relating to total social, emotional, and behavioral problems (Supplemental Appendix E), of which 15 evaluations were eligible for inclusion in the meta-analysis. Collectively, interventions demonstrated a small but significant effect for reducing problem behaviors in the short-term (*d* = −0.15, 95% CI [−0.28, −0.02]) (Figure 2). The meta-analysis of short-term outcomes included twenty effect sizes from eleven evaluations, with substantial between-studies heterogeneity (*I*^2^ = 62%). However, there was no evidence that interventions reduced total problem behaviors at longer-term follow-up (*d* = −0.07, 95% CI [−0.38, 0.25]) (Figure 2). For meta-analysis of longer-term outcomes, we included twelve effect sizes from six evaluations and observed moderate heterogeneity (*I*^2^ = 56%).

##### Total Social-Emotional Functioning and/or Impaired Functioning

Seventeen RCT evaluations of 14 interventions reported outcomes relating to social, emotional, and behavioral functioning and/or impaired functioning (Supplemental Appendix E), of which 16 evaluations of 13 interventions were eligible for inclusion in meta-analysis.

**Figure 3.**
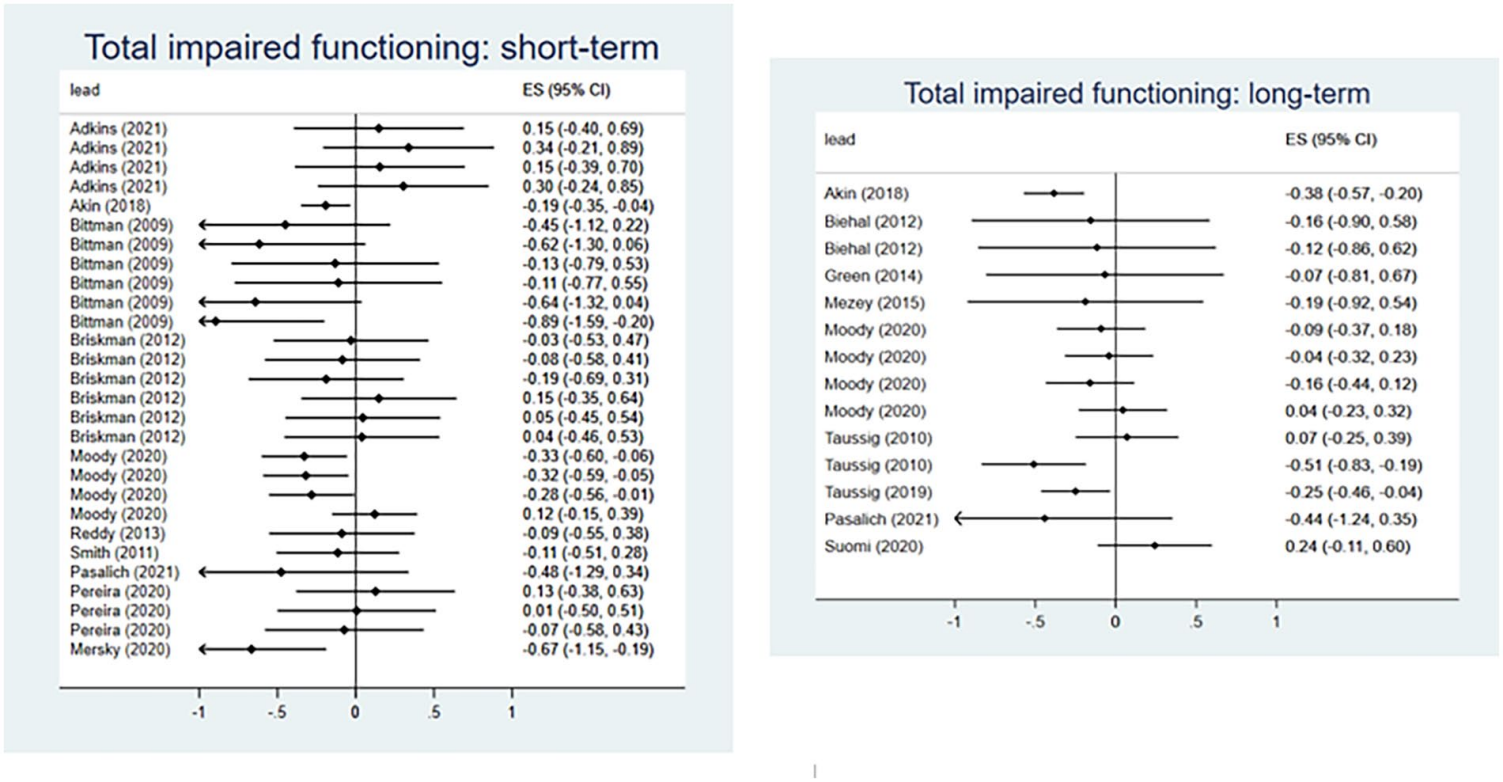
Meta-analysis forest plots for total social-emotional functioning: shorter-term and longer-term outcomes.

Collectively, interventions demonstrated a small but significant effect for reducing social-emotional functioning difficulties in the short-term (*d* = −0.18, 95% CI [−0.31, −0.05]) (Figure 3). The analysis included 28 effect sizes from ten evaluations, with moderate between-studies heterogeneity (*I*^2^ = 53%). When evaluated at longer-term follow-up, interventions showed some effect at reducing social-emotional functioning difficulties, with the effect approaching but not reaching statistical significance (*d* = −0.15, 95% CI [−0.40, 0.09]) (Figure 3). For longer-term follow-ups, we included 14 effect sizes from 8 evaluations and observed substantial heterogeneity (*I*^2^ = 63%).

##### Internalizing Behavior Problems

**Figure 4.**
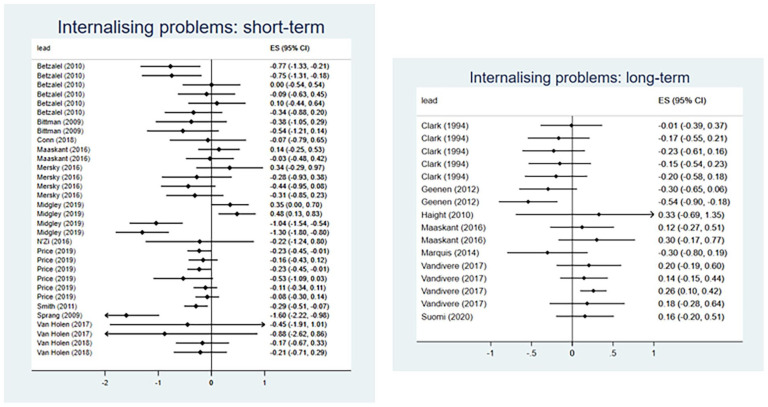
Meta-analysis forest plots for internalizing problem behaviors: short-term and long-term outcomes.

Nineteen evaluations of 19 interventions reported outcomes relating to internalizing behavior problems, of which 18 evaluations were eligible for inclusion in meta-analysis. Collectively, interventions demonstrated a medium effect for reducing internalizing problem behaviors in the short-term (*d* = −0.35, 95% CI [−0.61, −0.08]) (Figure 4). The analysis included 32 effect sizes from twelve evaluations, with substantial between-studies heterogeneity (*I*^2^ = 74%). However, there was no evidence that interventions reduced internalizing problem behaviors when evaluated at longer-term follow-up (*d* = −0.03, 95% CI [−0.31, 0.25]) (Figure 4). For longer-term follow-ups, we included 16 effect sizes from 7 evaluations and observed moderate heterogeneity (*I*^2^ = 53%).

##### Externalizing Behavior Problems

**Figure 5.**
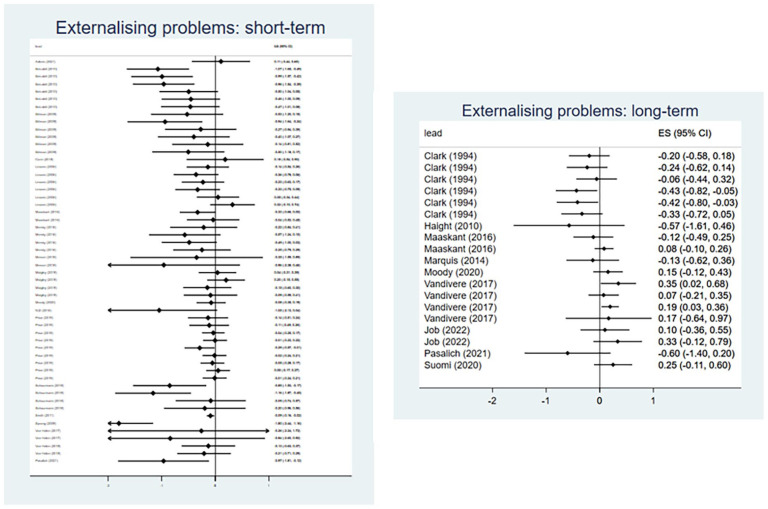
Meta-analysis forest plots for externalizing problem behaviors: short-term and long-term outcomes.

Twenty-seven evaluations of 26 interventions reported outcomes relating to externalizing behavior problems, of which 24 evaluations were eligible for inclusion in meta-analysis (Supplemental Appendix E). Collectively, interventions demonstrated a medium effect for reducing externalizing problem behaviors in the short-term (*d* = −0.30, 95% CI [−0.53, −0.08]) (Figure 5). The analysis included 54 effect sizes from 18 evaluations, with substantial between-studies heterogeneity (*I*^2^ = 73%). However, there was no evidence that interventions reduced externalizing problem behaviors when evaluated at longer-term follow-up (*d* = 0.02, 95% CI [−0.17, 0.20]) (Figure 5). For longer-term follow-ups, we included 19 effect sizes from 9 evaluations and observed moderate heterogeneity (*I*^2^ = 45%).

##### Anxiety and Depression

**Figure 6.**
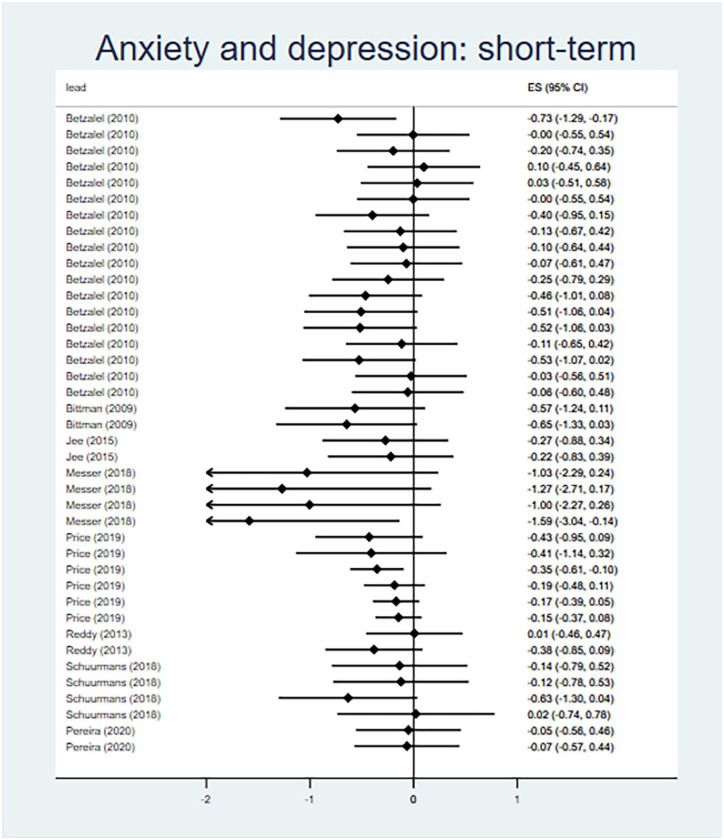
Meta-analysis forest plots for anxiety and depression: short-term outcomes.

Twelve evaluations of twelve interventions reported outcomes relating to anxiety and depression (Supplemental Appendix E), all of which were eligible for meta-analysis. Collectively, interventions demonstrated a medium effect for reducing scores on measures of anxiety and depression in the short-term (*d* = −0.26, 95% CI [−0.40, −0.13]), relative to control groups (Figure 6). The analysis included forty effect sizes from eight evaluations, with minimal between-studies heterogeneity (*I*^2^ = 8%). There were insufficient available effect sizes to conduct a meta-analysis of long-term (>6 months follow-up) intervention effects.

##### Other Outcome Domains

Four evaluations of three interventions assessed stress, post-traumatric stress disorder (PTSD) and trauma (Supplemental Appendix E), with evidence of fewer dissociative symptoms at 6-month follow up in a 2010 (*d* = −0.29, 95% CI [−0.50, −0.09]) and a linked 2019 evaluation of Fostering Healthy Futures (Taussig & Culhane, 2010; Taussig et al., 2019), but no group differences in post-traumatic symptoms. One evaluation assessed child attachment disorder, with no evidence of effectiveness at 9-month follow-up (Minnis et al., 2001). Two evaluations of two interventions reported outcomes for children and young people’s attention and hyperactivity. Neither intervention demonstrated effectiveness (Adkins et al., 2021; Moody et al., 2020). For each of these domains, there was an insufficient number of effect sizes to conduct meta-analysis.

#### Self-harm and Suicide

Two intervention evaluations measured self-harm and suicide-related outcomes (suicide attempt, suicidal-ideation, self-harm incidents), but neither reported any significant group differences (Bittman et al., 2009; Mezey et al., 2015). There were insufficient data to conduct a meta-analysis for this outcome domain.

### GRADE Assessment of Certainty

GRADE assessments are shown in Supplemental Appendix F. For short-term outcomes, we assessed there to be low certainty of effectiveness for interventions targeting total problem behaviors, social-emotional functioning, and externalizing problems. For each of these domains, we assessed a serious risk of bias and serious imprecision. All other outcome domains were assessed as being of very low certainty of evidence, based on serious risk of bias and very serious imprecision. All long-term outcome domains were assessed as having very low certainty of effectiveness due to the same risk of bias and serious imprecision concerns.

## Discussion

In the CHIMES systematic review, we synthesized evidence of effectiveness for interventions targeting the mental health and wellbeing of care-experienced children and young people, as evaluated via an RCT study design. Included interventions primarily operated at the intrapersonal and interpersonal level, focusing on the social and emotional competencies of young people, supporting the development of carer knowledge, skills and self-efficacy, and facilitating positive relationships between young people and significant others.

While the existing evidence-base tends to synthesize evidence for a limited range of outcomes, the current meta-analysis found that interventions positively supported a range of mental health-related outcomes, namely: total social, emotional, and behavioral problems; social-emotional functioning; externalizing problem behaviors; internalizing problem behaviors; and depression and anxiety. However, these effects were only observed where post-baseline measurements were assessed within 6 months. For post-baseline measurements for more than 6 months, no significant positive impacts were detected. As existing reviews of interventions for this population have tended to aggregate findings across follow-up intervals, and have not conducted meta-analysis according to length to follow-up, it is not known if the review findings reflect the wider evidence-base.

Although it is important not to speculate why there are differences between shorter-term and longer-term outcome measurements, there are a couple of areas that would be expedient to explore further. Firstly, the underpinning mechanisms of change in existing interventions may not be sufficient to generate long-term change. This may be particularly true of brief interventions with longer-term follow-up, where participants may be unsupported in the sustained enaction of change (e.g., application of new parenting techniques) and efforts to change are inhibited by entrenched and seemingly intractable system structures that reinforce the status quo (e.g., culturally endorsed parenting norms) (Kirton & Thomas, 2011; Lotty et al., 2020). This reflects wider concerns within intervention research: that approaches are often minimally disruptive and do not sufficiently target structural drivers of the problem or reconfigure contextual features to support change (Hawe, 2015; Moore et al., 2018). Second, for longer interventions with long-term follow-up, there may be issues around implementation. As a result, participants may not receive the intervention as intended. For example, process evaluations of brief interventions that provide concentrated training to carers show that interventions are often well attended with high levels of fidelity and acceptability (Evans, Trubey et al., 2023). More complex and prolonged interventions can be hampered by resource issues and the fact that care-experienced individuals denote a somewhat transient population (Evans, Trubey et al., 2023; Mezey et al., 2015).

There are a range of limitations with the evidence-base. In terms of scope, there is a clear lack of interventions targeting wellbeing and suicide-related outcomes. This paucity has been identified with more widely, with limited approaches for those involved in child protection services (Russell et al., 2021). Interventions to target these outcomes are imperative, as research indicates that care-experienced populations report relatively pronounced adversity when compared to non-care-experienced peers (Bronsard et al., 2016; Engler et al., 2022; Ford et al., 2018; Long et al., 2017).

Included intervention evaluations were also characterized by major methodological limitations, with significant risk of bias noted in many instances. Quality appraisal identified key issues, including “risk of bias from measurement of outcomes,” with outcomes typically self-reported by children and young people or reported by carers (or in some instances teachers or clinicians) who were unblinded to group allocations. In several instances, there were notable discrepancies between children and young people’s self-reported outcomes and the same outcomes as reported by adults.

### Review Limitations

The review has several limitations that should be considered when interpreting the findings. First, to support meta-analysis, follow-up time from baseline was categorized as 6 months and less or more than 6 months. Within each time frame, interventions delivery ranged from a few days to a few months. This meant that in some instances follow-up outcomes were measured immediately post-intervention and in other cases they were measured after a significant period following completion of intervention. As such, it is not possible to clearly ascertain if the lack of long-term effectiveness was linked to intervention duration. The trend for beneficial short-term effects for some outcomes may also have been influenced by studies measuring outcomes immediately post-intervention—although examination of the data suggests that studies reporting significant short-term effectiveness varied in follow-up time. Due to the diversity of intervention length and limited availability of data on intervention length, it was not possible to conduct further meta-analysis by duration—we cannot rule out the possibility, therefore, that intervention duration was an important component for driving short-term effectiveness. Second, evaluations tended to report group outcomes for the whole study sample, and it was not possible to disaggregate data for participants according to gender, ethnicity, and placement types, which are known predictors of mental health (Kennedy et al., 2022). Progress in assessing outcomes for diverse groups in primary evaluations would allow future reviews to look at variation in effectiveness within the heterogeneous care-experienced population.

### Implications for Future Research

The review has several possible implications for future research. There is an evident need to develop and evaluate interventions that target subjective wellbeing and suicide-related outcomes. For all intervention development in this area, there needs to a clear focus on understanding the mechanisms through which interventions will bring about change, how change may be sustained over a longer period, and how contextual factors may inhibit or facilitate this process (Skivington et al., 2021). Such interventions need to be responsive to local context and their intended populations and should be developed with input and support from children and young people and other key stakeholders.

Centrally, there needs to be an improvement in the quality of evaluation of interventions for care-experienced populations. Conduct of RCTs have been well documented, with reported issues regarding randomization and contamination of intervention arms (Mezey et al., 2015). Recruiting and retaining participants from this population, particularly over longer-term follow-up periods, is always likely to be challenging. Regardless, there needs to be further work to anticipate and mitigate such risks of bias. Use of key methodological frameworks such as the Medical Research Council guidance for developing and evaluating interventions be supportive in ensuring a high-quality study design (Skivington et al., 2021). Improved reporting of interventions and their associated evaluations, structured with the use of key reporting guidelines, will also help strengthen future systematic reviews (Schulz et al., 2010).

### Recommendations for Policy and Practice

There is currently some evidence that interpersonal and intrapersonal interventions can have some short-term beneficial effects on care-experienced children and young people’s mental, behavioral, and neurodevelopmental outcomes. However, policy makers and practitioners need to be conscious that care-experienced children and young people will likely need ongoing, structural support to sustain the effects of relatively short-term interventions. There is a pressing need for broader interventions in this area that target organizational, community, or policy-level change, and which may be more successful in supporting and sustaining long-term change. Such interventions will likely require significant investment of resources, and collaboration between policy makers and practitioners.

## Conclusions

The CHIMES review and meta-analysis identified that available interventions report some positive impacts on mental, behavioral, and neurodevelopmental disorders in the short term, but these are not achieved in the longer term. In both cases, certainty of effectiveness is constrained by methodological limitations including risk of bias and imprecision. There was a lack of available data for subjective wellbeing and suicide-related outcomes, with individual interventions reporting limited effectiveness. Future research requires more robust evaluation and further exploration as to why interventions may struggle to achieve effects when delivered and evaluated over a longer time-frame.

**Table:** 

Critical Findings
• We identified 35 unique interventions evaluated by randomized controlled trials aimed at improving outcomes for children and young people in care—primarily evaluated in the United States or United Kingdom • The current landscape is characterized by interventions operating at an interpersonal or intra-personal level—with very few interventions targeting organizational, community or policy-level change • There is some evidence that existing interventions have beneficial effects on children and young people’s mental, behavioral, or neurodevelopmental outcomes in the short term (up to 6 months after interventions commence) • However, there is currently very little evidence that these benefits are sustained beyond 6 months follow-up • There are several methodological limitations with evaluations—including risk of bias and imprecision—that limit certainty in the effectiveness of current interventions
Implications for Practice, Policy, and Research
• Policy makers and practitioners need to be conscious about the needs of children and young people in care—and their carers—there needs to be ongoing, structural support to sustain the effects of relatively short-term interventions • There is a pressing need for interventions in this area that target organizational, community, or policy-level change, and which may be more successful in supporting and sustaining long-term change. Such interventions will likely require significant investment of resources and collaboration between policy-makers and practitioners. • Evaluations of interventions are currently focused narrowly on outcomes relating to behavioral outcomes, with some mental health and neurodevelopmental outcomes—evaluations that assess a wider range of child-level outcomes, including subjective well-being and self-harm and suicide-related outcomes, are needed • Use of methodological frameworks, and improved reporting of interventions and their associated evaluations, will strengthen future systematic reviews and the existing evidence-base for interventions in this area

## Supplemental Material

sj-docx-1-tva-10.1177_15248380241227987 – Supplemental material for Effectiveness of Mental Health and Wellbeing Interventions for Children and Young People in Foster, Kinship, and Residential Care: Systematic Review and Meta-Analysis Supplemental material, sj-docx-1-tva-10.1177_15248380241227987 for Effectiveness of Mental Health and Wellbeing Interventions for Children and Young People in Foster, Kinship, and Residential Care: Systematic Review and Meta-Analysis by Rob Trubey, Rhiannon Evans, Sarah McDonald, Jane Noyes, Mike Robling, Simone Willis, Maria Boffey, Charlotte Wooders, Soo Vinnicombe and G. J. Melendez-Torres in Trauma, Violence, & Abuse

sj-docx-2-tva-10.1177_15248380241227987 – Supplemental material for Effectiveness of Mental Health and Wellbeing Interventions for Children and Young People in Foster, Kinship, and Residential Care: Systematic Review and Meta-Analysis Supplemental material, sj-docx-2-tva-10.1177_15248380241227987 for Effectiveness of Mental Health and Wellbeing Interventions for Children and Young People in Foster, Kinship, and Residential Care: Systematic Review and Meta-Analysis by Rob Trubey, Rhiannon Evans, Sarah McDonald, Jane Noyes, Mike Robling, Simone Willis, Maria Boffey, Charlotte Wooders, Soo Vinnicombe and G. J. Melendez-Torres in Trauma, Violence, & Abuse

sj-docx-3-tva-10.1177_15248380241227987 – Supplemental material for Effectiveness of Mental Health and Wellbeing Interventions for Children and Young People in Foster, Kinship, and Residential Care: Systematic Review and Meta-Analysis Supplemental material, sj-docx-3-tva-10.1177_15248380241227987 for Effectiveness of Mental Health and Wellbeing Interventions for Children and Young People in Foster, Kinship, and Residential Care: Systematic Review and Meta-Analysis by Rob Trubey, Rhiannon Evans, Sarah McDonald, Jane Noyes, Mike Robling, Simone Willis, Maria Boffey, Charlotte Wooders, Soo Vinnicombe and G. J. Melendez-Torres in Trauma, Violence, & Abuse

sj-docx-4-tva-10.1177_15248380241227987 – Supplemental material for Effectiveness of Mental Health and Wellbeing Interventions for Children and Young People in Foster, Kinship, and Residential Care: Systematic Review and Meta-Analysis Supplemental material, sj-docx-4-tva-10.1177_15248380241227987 for Effectiveness of Mental Health and Wellbeing Interventions for Children and Young People in Foster, Kinship, and Residential Care: Systematic Review and Meta-Analysis by Rob Trubey, Rhiannon Evans, Sarah McDonald, Jane Noyes, Mike Robling, Simone Willis, Maria Boffey, Charlotte Wooders, Soo Vinnicombe and G. J. Melendez-Torres in Trauma, Violence, & Abuse

sj-docx-5-tva-10.1177_15248380241227987 – Supplemental material for Effectiveness of Mental Health and Wellbeing Interventions for Children and Young People in Foster, Kinship, and Residential Care: Systematic Review and Meta-Analysis Supplemental material, sj-docx-5-tva-10.1177_15248380241227987 for Effectiveness of Mental Health and Wellbeing Interventions for Children and Young People in Foster, Kinship, and Residential Care: Systematic Review and Meta-Analysis by Rob Trubey, Rhiannon Evans, Sarah McDonald, Jane Noyes, Mike Robling, Simone Willis, Maria Boffey, Charlotte Wooders, Soo Vinnicombe and G. J. Melendez-Torres in Trauma, Violence, & Abuse

sj-docx-6-tva-10.1177_15248380241227987 – Supplemental material for Effectiveness of Mental Health and Wellbeing Interventions for Children and Young People in Foster, Kinship, and Residential Care: Systematic Review and Meta-Analysis Supplemental material, sj-docx-6-tva-10.1177_15248380241227987 for Effectiveness of Mental Health and Wellbeing Interventions for Children and Young People in Foster, Kinship, and Residential Care: Systematic Review and Meta-Analysis by Rob Trubey, Rhiannon Evans, Sarah McDonald, Jane Noyes, Mike Robling, Simone Willis, Maria Boffey, Charlotte Wooders, Soo Vinnicombe and G. J. Melendez-Torres in Trauma, Violence, & Abuse
