# Supplementary material for: Effectiveness of Mental Health and Wellbeing Interventions for Children and Young People in Foster, Kinship, and Residential Care: Systematic Review and Meta-Analysis
Source: Trauma Violence Abuse. 2024 Feb 16;25(4):2829–44. doi: 10.1177/15248380241227987 (PMC11370152; doi:10.1177/15248380241227987)
Supplement: sj-docx-2-tva-10.1177_15248380241227987 – Supplemental material for Effectiveness of Mental Health and Wellbeing Interventions for Children and Young People in Foster, Kinship, and Residential Care: Systematic Review and Meta-Analysis [file sj-docx-2-tva-10.1177_15248380241227987.docx]

**Appendix B: Studies excluded from meta-analysis**

| **Intervention** | **Country** | **Target population** | **Intervention duration** | **Children and young people’s age** | **Reason for excluding from meta-analysis** |
| --- | --- | --- | --- | --- | --- |
|  |  |  |  |  |  |
| Attachment and Biobehavioural Catchup (ABC), Dozier (2006) | USA | Multiple | 8-16 weeks | Infants / pre-school (0-5) | Did not present outcome data in an extractable format. |
| Head Start, Lipscomb (2013) | USA | Multiple | 20+ weeks | Infants / pre-school (0-5) | No relative comparative outcome data were reported. |
| Multidimensional Treatment Foster Care (MTFC), Jonkman (2017) | Netherlands | Multiple | 20+ weeks | Younger children (6-11) | Describes results of an RCT and quasi-experimental design evaluation, but results are not presented separately – so functionally a quasi-experimental design. |
| Supporting Looked After Children and Care Leavers In Decreasing Drugs (SOLID), Alderson 2020 | UK | Children & young people | 8-16 weeks | Older adolescents (16+) | Reported dropout rate was too high for outcome data to be a valid estimate of effectiveness. |
