# Supplementary material for: Effectiveness of Mental Health and Wellbeing Interventions for Children and Young People in Foster, Kinship, and Residential Care: Systematic Review and Meta-Analysis
Source: Trauma Violence Abuse. 2024 Feb 16;25(4):2829–44. doi: 10.1177/15248380241227987 (PMC11370152; doi:10.1177/15248380241227987)
Supplement: sj-docx-3-tva-10.1177_15248380241227987 – Supplemental material for Effectiveness of Mental Health and Wellbeing Interventions for Children and Young People in Foster, Kinship, and Residential Care: Systematic Review and Meta-Analysis [file sj-docx-3-tva-10.1177_15248380241227987.docx]

**Appendix C: Summary of included RCT evaluations and interventions**

| **Intervention** | **Socio-ecological domain** | | | | | **Evaluation design** | **Country** | **Target population** | **Intervention duration** | **CYP age** | **Outcome domains** | | |
| --- | --- | --- | --- | --- | --- | --- | --- | --- | --- | --- | --- | --- | --- |
|  | **Interpersonal** | **Intrapersonal** | **Policy** | **Community** | **Organisational** |  |  |  |  |  | **Wellbeing** | **Mental health** | **Self-harm / suicide** |
| Attachment and Biobehavioural Catchup (ABC), Dozier (2006) | √ | √ |  |  |  | RCT | USA | Multiple | 8-16 weeks | Infants / pre-school (0-5) |  | √ |  |
| Attachment and Biobehavioural Catchup (ABC), Sprang (2009) | √ | √ |  |  |  | RCT | USA | Multiple | 8-16 weeks | Infants / pre-school (0-5) |  | √ |  |
| Child Adult Relationship Enhancement (CARE), Messer (2018) | √ |  |  |  |  | RCT | USA | Foster carers | 2-7 weeks | Younger children (6-11) |  | √ |  |
| Child Directed Interaction Training (CDIT), N'Zi (2016) | √ |  |  |  |  | RCT | USA | Multiple | 2-7 weeks | Younger children (6-11) |  | √ |  |
| Cognitive and Affective Bibliotherapy, Betzalel (2010) |  | √ |  |  |  | RCT | Israel | Children & young people | 8-16 weeks | Young adolescents (12-16) |  | √ |  |
| Cognitively-Based Compassion Training (CBCT), Reddy (2013) |  | √ |  |  |  | RCT | USA | Children & young people | 2-7 weeks | Young adolescents (12-16) |  | √ |  |
| Communication and Attachment Training for Foster Carers, Minnis (2001) | √ |  |  |  |  | RCT | UK | Foster carers | 2-7 weeks | Younger children (6-11) |  | √ |  |
| Connect-KP, Pasalich (2021) | √ |  |  |  |  | RCT | Australia | Foster carers | 8-16 weeks | Younger children (6-11) |  | √ |  |
| Dojo: Biofeedback videogame, Schuurmans (2018) |  | √ |  |  |  | RCT | Netherlands | Children & young people | 8-16 weeks | Young adolescents (12-16) |  | √ |  |
| Family Finding, Vandivere (2017) | √ |  |  |  |  | RCT | USA | Children & young people | 20+ weeks | Young adolescents (12-16) |  | √ |  |
| Family Minds, Adkins (2021) | √ |  |  |  |  | RCT | USA | Foster carers | 2-7 weeks | Younger children (6-11) |  | √ |  |
| Foster carer and foster children group-based intervention, Smith (2011) | √ |  |  |  |  | RCT | USA | Multiple | 2-7 weeks | Younger children (6-11) |  | √ |  |
| Foster Parent Intervention, Van Holen (2017) | √ |  |  |  |  | RCT | Belgium | Foster carers | 8-16 weeks | Younger children (6-11) |  | √ |  |
| Fostering Changes (FC), Briskman (2012) | √ |  |  |  |  | RCT | UK | Foster carers | 8-16 weeks | Younger children (6-11) |  | √ |  |
| Fostering Changes (FC), Moody (2020) | √ |  |  |  |  | RCT | UK | Multiple | 8-16 weeks | Younger children (6-11) |  | √ |  |
| Fostering Healthy Futures (FHF), Taussig (2010) | √ | √ |  |  |  | RCT | USA | Children & young people | 20+ weeks | Younger children (6-11) | √ | √ |  |
| Fostering Healthy Futures (FHF), Taussig (2019) | √ | √ |  |  |  | RCT | USA | Children & young people | 20+ weeks | Younger children (6-11) | √ | √ |  |
| Fostering Individualised Assistance Program (FIAP), Clark (1994) |  | √ |  | √ | √ | RCT | USA | Multiple | 20+ weeks | Younger children (6-11) |  | √ |  |
| Head Start, Lipscomb (2013) |  |  | √ |  |  | RCT | USA | Multiple | 20+ weeks | Infants / pre-school (0-5) |  | √ |  |
| HealthRHYTHMS Bittman (2009) | √ | √ |  |  |  | RCT | USA | Children & young people | 2-7 weeks | Young adolescents (12-16) |  | √ | √ |
| Incredible Years (IY), Conn (2018) | √ |  |  |  |  | RCT | USA | Foster carers | 8-16 weeks | Infants / pre-school (0-5) |  | √ |  |
| Incredible Years (IY), Linares (2006) | √ |  |  |  |  | RCT | USA | Foster carers | 8-16 weeks | Younger children (6-11) |  | √ |  |
| kConnect, Suomi (2020) | √ |  |  |  |  | RCT | Australia | Multiple | 20+ weeks | Younger children (6-11) |  | √ |  |
| Keeping foster and kinship carers supported (KEEP), Chamberlain (2008) | √ |  |  |  |  | RCT | USA | Multiple | 8-16 weeks | Younger children (6-11) |  | √ |  |
| Keeping foster and kinship carers supported (KEEP), Price (2015) | √ |  |  |  |  | RCT | USA | Multiple | 8-16 weeks | Younger children (6-11) |  | √ |  |
| Keeping foster and kinship carers supported (KEEP), Price (2019) | √ |  |  |  |  | RCT | USA | Multiple | 8-16 weeks | Younger children (6-11) |  | √ |  |
| Life Story, Haight (2010) | √ | √ |  |  |  | RCT | USA | Children & young people | 20+ weeks | Younger children (6-11) |  | √ |  |
| Mentalization‐based therapy (MBT), Midgley (2019) | √ | √ |  |  |  | RCT | UK | Multiple | 8-16 weeks | Younger children (6-11) |  | √ |  |
| Mentoring intervention for teenage pregnancy, Mezey (2015) | √ |  |  |  |  | RCT | UK | Children & young people | 20+ weeks | Older adolescents (16+) |  | √ | √ |
| Mindfulness, Jee (2015) |  | √ |  |  |  | RCT | USA | Children & young people | 8-16 weeks | Older adolescents (16+) |  | √ |  |
| Multidimensional Treatment Foster Care (MTFC), Biehal (2012) | √ |  |  |  |  | RCT | UK | Multiple | 20+ weeks | Young adolescents (12-16) |  | √ |  |
| Multidimensional Treatment Foster Care (MTFC), Green (2014) | √ |  |  |  |  | RCT | UK | Children & young people | 20+ weeks | Young adolescents (12-16) |  | √ |  |
| Multidimensional Treatment Foster Care (MTFC), Jonkman (2017) | √ |  |  |  |  | RCT | Netherlands | Multiple | 20+ weeks | Younger children (6-11) |  | √ |  |
| Nonviolent Resistance (NVR) Training, Van Holen (2018) | √ |  |  |  |  | RCT | Belgium | Foster carers | 8-16 weeks | Young adolescents (12-16) |  | √ |  |
| Parent Management Training Oregon (PMTO), Akin (2018) | √ |  |  |  |  | RCT | USA | Biological parents | 20+ weeks | Younger children (6-11) |  | √ |  |
| Parent Management Training Oregon (PMTO), Maaskant (2016) | √ |  |  |  |  | RCT | Netherlands | Multiple | 20+ weeks | Younger children (6-11) |  | √ |  |
| Parent-child Interaction Therapy (PCIT), Mersky (2016) | √ |  |  |  |  | RCT | USA | Multiple | 8-16 weeks | Infants / pre-school (0-5) |  | √ |  |
| Parent-Child Interaction Therapy (PCIT) - Brief + Extended, Mersky (2020) | √ |  |  |  |  | RCT | USA | Multiple | 8-16 weeks | Infants / pre-school (0-5) |  | √ |  |
| Pathways Home, DeGarmo (2013) | √ |  |  |  |  | RCT | USA | Multiple | 20+ weeks | Younger children (6-11) |  | √ |  |
| Supporting Looked After Children and Care Leavers In Decreasing Drugs (SOLID), Alderson 2020 | √ |  |  |  |  | RCT | UK | Children & young people | 8-16 weeks | Older adolescents (16+) |  | √ |  |
| Take Charge, Geenen (2012) |  | √ |  |  |  | RCT | USA | Children & young people | 20+ weeks | Young adolescents (12-16) |  | √ |  |
| Teach Your Children Well, Marquis (2014) | √ | √ |  |  |  | RCT | Canada | Children & young people | 20+ weeks | Younger children (6-11) |  | √ |  |
| Triple P for Foster Parents, Job (2022) | √ |  |  |  |  | RCT | Germany | Multiple | 2-7 weeks | Infants / pre-school (0-5) |  | √ |  |
| Wave by Wave Surf Therapy, Pereira (2020) | √ | √ |  |  |  | RCT | Portugal | Children & young people | 20+ weeks | Young adolescents (12-16) | √ | √ |  |
