# Supplementary material for: Effectiveness of Mental Health and Wellbeing Interventions for Children and Young People in Foster, Kinship, and Residential Care: Systematic Review and Meta-Analysis
Source: Trauma Violence Abuse. 2024 Feb 16;25(4):2829–44. doi: 10.1177/15248380241227987 (PMC11370152; doi:10.1177/15248380241227987)
Supplement: sj-docx-4-tva-10.1177_15248380241227987 – Supplemental material for Effectiveness of Mental Health and Wellbeing Interventions for Children and Young People in Foster, Kinship, and Residential Care: Systematic Review and Meta-Analysis [file sj-docx-4-tva-10.1177_15248380241227987.docx]

**Appendix D: Quality Appraisal of Outcome Evaluations**

| Intervention | Author | Bias from randomisation | Bias arising from deviations from protocol | Bias arising from missing data | Bias arising from outcome measurement | Bias arising from selection of reported outcomes | Overall risk of bias |
| --- | --- | --- | --- | --- | --- | --- | --- |
| Attachment and Biobehavioural Catchup (ABC) | Dozier (2006) | Low risk | Low risk | Low risk | Some concerns | Low risk | Some concerns |
| Attachment and Biobehavioural Catchup (ABC) | Sprang (2009) | Low risk | Low risk | Low risk | Some concerns | Low risk | Some concerns |
| Child Adult Relationship Enhancement (CARE) | Messer (2018) | Some concerns | Low risk | Low risk | Some concerns | Low risk | Some concerns |
| Child Directed Interaction Training (CDIT) | N'Zi (2016) | Low risk | Low risk | Low risk | Some concerns | Low risk | Some concerns |
| Cognitive and Affective Bibliotherapy | Betzalel (2010) | Some concerns | Low risk | Low risk | Some concerns | Low risk | Some concerns |
| Cognitively-Based Compassion Training (CBCT) | Reddy (2013) | Low risk | Low risk | Some concerns | Some concerns | Low risk | Some concerns |
| Communication and Attachment Training for Foster Carers | Minnis (2001) | Low risk | Low risk | Low risk | Some concerns | Low risk | Some concerns |
| Connect-KP | Pasalich (2021) | Low risk | Low risk | Low risk | Some concerns | Low risk | Some concerns |
| Dojo: Biofeedback videogame | Schuurmans (2018) | Low risk | Low risk | Low risk | Some concerns | Low risk | Some concerns |
| Family Finding | Vandivere (2017) | Low risk | Low risk | Some concerns | Some concerns | Low risk | Some concerns |
| Family Minds | Adkins (2021) | Low risk | Low risk | Low risk | Some concerns | Low risk | Some concerns |
| Foster carer and foster children group-based intervention | Smith (2011) | Low risk | Low risk | Low risk | Some concerns | Low risk | Some concerns |
| Foster Parent Intervention | Van Holen (2017) | Low risk | Low risk | Low risk | Some concerns | Low risk | Some concerns |
| Fostering Changes (FC) | Briskman (2012) | Low risk | Low risk | Low risk | Some concerns | Low risk | Some concerns |
| Fostering Changes (FC) | Moody (2020) | Low risk | Low risk | Low risk | Some concerns | Low risk | Some concerns |
| Fostering Healthy Futures (FHF) | Taussig (2010) | Low risk | Low risk | Low risk | Some concerns | Low risk | Some concerns |
| Fostering Healthy Futures (FHF) | Taussig (2019) | Low risk | Low risk | Low risk | Some concerns | Low risk | Some concerns |
| Fostering Individualised Assistance Program (FIAP) | Clark (1994) | Low risk | Low risk | Low risk | Some concerns | Low risk | Some concerns |
| Head Start | Lipscomb (2013) | Some concerns | Low risk | Some concerns | Some concerns | Low risk | High risk |
| HealthRHYTHMS | Bittman (2009) | Low risk | Low risk | Low risk | Some concerns | Low risk | Some concerns |
| Incredible Years (IY) | Conn (2018) | Some concerns | Low risk | Low risk | Some concerns | Low risk | Some concerns |
| Incredible Years (IY) | Linares (2006) | Low risk | Low risk | Low risk | Some concerns | Low risk | Some concerns |
| kConnect | Suomi (2020) | Low risk | Some concerns | Some concerns | Some concerns | Low risk | High risk |
| Keeping foster and kinship carers supported (KEEP) | Chamberlain (2008) | Low risk | Low risk | Low risk | Some concerns | Low risk | Some concerns |
| Keeping foster and kinship carers supported (KEEP) | Price (2015) | Low risk | Low risk | Low risk | Some concerns | Low risk | Some concerns |
| Keeping foster and kinship carers supported (KEEP) | Price (2019) | Some concerns | Low risk | Low risk | Some concerns | Low risk | Some concerns |
| Life Story | Haight (2010) | Low risk | Low risk | Some concerns | Some concerns | Low risk | Some concerns |
| Mentalization‐based therapy (MBT) | Midgley (2019) | Low risk | Low risk | Low risk | Some concerns | Low risk | Some concerns |
| Mentoring intervention for teenage pregnancy | Mezey (2015) | Low risk | High risk | Some concerns | Some concerns | Low risk | High risk |
| Mindfulness | Jee (2015) | Low risk | Low risk | Low risk | Some concerns | Low risk | Some concerns |
| Multidimensional Treatment Foster Care (MTFC) | Biehal (2012) | High risk | High risk | Some concerns | Some concerns | Low risk | High risk |
| Multidimensional Treatment Foster Care (MTFC) | Green (2014) | Low risk | Low risk | Some concerns | Some concerns | Low risk | Some concerns |
| Multidimensional Treatment Foster Care (MTFC) | Jonkman (2017) | Low risk | Low risk | Low risk | Some concerns | Low risk | Some concerns |
| Nonviolent Resistance (NVR) Training | Van Holen (2018) | Low risk | Low risk | Low risk | Some concerns | Low risk | Some concerns |
| Parent Management Training Oregon (PMTO) | Akin (2018) | Low risk | Low risk | Low risk | Some concerns | Low risk | Some concerns |
| Parent Management Training Oregon (PMTO) | Akin (2019) | Low risk | Some concerns | Low risk | Some concerns | Low risk | Some concerns |
| Parent-Child Interaction Therapy (PCIT) | Mersky (2016) | Low risk | Low risk | Low risk | Some concerns | Low risk | Some concerns |
| Parent-Child Interaction Therapy (PCIT) | Mersky (2020) | Low risk | Low risk | Low risk | Some concerns | Low risk | Some concerns |
| Pathways Home | DeGarmo (2013) | Low risk | Low risk | Low risk | Some concerns | Low risk | Some concerns |
| Supporting Looked After Children and Care Leavers In Decreasing Drugs (SOLID) | Alderson (2020) | Low risk | Some concerns | High risk | Some concerns | Low risk | High risk |
| Take Charge | Geenen (2012) | Low risk | Low risk | Low risk | Some concerns | Some concerns | Some concerns |
| Teach Your Children Well | Marquis (2014) | Low risk | Some concerns | Low risk | Some concerns | Low risk | Some concerns |
| Triple P for Foster Parents | Job (2022) | Some concerns | Some concerns | Some concerns | Some concerns | Low risk | High risk |
| Wave by Wave Surf Therapy | Pereira (2020) | Low risk | Low risk | Some concerns | Some concerns | Low risk | Some concerns |
