# Supplementary material for: Effectiveness of Mental Health and Wellbeing Interventions for Children and Young People in Foster, Kinship, and Residential Care: Systematic Review and Meta-Analysis
Source: Trauma Violence Abuse. 2024 Feb 16;25(4):2829–44. doi: 10.1177/15248380241227987 (PMC11370152; doi:10.1177/15248380241227987)
Supplement: sj-docx-5-tva-10.1177_15248380241227987 – Supplemental material for Effectiveness of Mental Health and Wellbeing Interventions for Children and Young People in Foster, Kinship, and Residential Care: Systematic Review and Meta-Analysis [file sj-docx-5-tva-10.1177_15248380241227987.docx]

## Appendix E: Effect size tables for all outcome domains

#### Quality of life

| **Intervention** | **Lead author** | **Int. length (weeks)** | **Outcome assessed** | **Tool** | **Informant** | **Data type** | **Follow-up** | **Follow-up (category)** | **Effect size** | **95% CI (low)** | **95% CI (high)** |
| --- | --- | --- | --- | --- | --- | --- | --- | --- | --- | --- | --- |
| Wave by Wave Surf Therapy | Pereira (2020) | 21 | Health-related quality of life | Kidscreen-10 | Child/young person | Scale | Post-test | 4-6 months | 0.25 | -0.26 | 0.76 |
| Fostering Healthy Futures (FHF) | Taussig (2010) | 30 | Quality of Life | LSS | Child/young person | Scale | Post intervention | 7-12 months | 0.42 | 0.12 | 0.71 |
| Fostering Healthy Futures (FHF) | Taussig (2010) | 30 | Quality of Life | LSS | Child/young person | Scale | 6 month post-intervention | 13-18 months | 0.14 | -0.17 | 0.45 |
| Fostering Healthy Futures (FHF) | Taussig (2019) | 30 | Quality of Life | LSS | Child/young person | Scale | 6 month post-intervention | 13-18 months | 0.16 | -0.04 | 0.36 |

#### Total social, emotional, and behavioural problems

| **Intervention** | **Lead author** | **Int. length (weeks)** | **Outcome assessed** | **Tool** | **Informant** | **Data type** | **Follow-up** | **Follow-up (category)** | **Effect size** | **95% CI (low)** | **95% CI (high)** |
| --- | --- | --- | --- | --- | --- | --- | --- | --- | --- | --- | --- |
| Attachment and Biobehavioural Catchup (ABC) | Dozier (2006) | 10 | Problem behaviour | PDR | Parent/carer | Scale | 1-month follow up | 0-3 months | *Insufficient data to calculate effect size* | | |
| Cognitive and Affective Bibliotherapy - Affective | Betzalel (2010) | 8 | Total problems | TRF | Clinician | Scale | Follow-up | 4-6 months | -1.02 | -1.60 | -0.44 |
| Cognitive and Affective Bibliotherapy - Cognitive | Betzalel (2010) | 8 | Total problems | TRF | Clinician | Scale | Follow-up | 4-6 months | -0.21 | -0.75 | 0.33 |
| Communication and Attachment Training for Foster Carers | Minnis (2001) | 3 | Total problems | SDQ | Teacher | Scale | 9 month follow up | 7-12 months | 0.27 | -0.06 | 0.60 |
| Communication and Attachment Training for Foster Carers | Minnis (2001) | 3 | Total problems | SDQ | Parent/carer | Scale | 9 month follow up | 7-12 months | -0.10 | -0.43 | 0.23 |
| Communication and Attachment Training for Foster Carers | Minnis (2001) | 3 | Total problems | SDQ | Child/young person | Scale | 9 month follow up | 7-12 months | -0.28 | -0.61 | 0.04 |
| Family Finding | Vandivere (2017) | 20 | Total problems - clinical level | YSR | Child/young person | Categorical | 12 month follow up | 7-12 months | 0.27 | -0.06 | 0.60 |
| Family Finding | Vandivere (2017) | 20 | Total problems - borderline or clinical level | YSR | Child/young person | Categorical | 12 month follow up | 7-12 months | 0.11 | -0.18 | 0.40 |
| Family Finding | Vandivere (2017) | 20 | Total problems - borderline or clinical level | YSR | Child/young person | Categorical | 24 month follow up | 19-24 months | 0.17 | -0.49 | 0.83 |
| Family Finding | Vandivere (2017) | 20 | Total problems - clinical level | YSR | Child/young person | Categorical | 24 month follow up | 19-24 months | 0.20 | -0.65 | 1.06 |
| Fostering Changes (FC) | Briskman (2012) | 12 | Total carer-defined problems | The Carer-Defined Problems Scale | Parent/carer | Scale | Post intervention | 0-3 months | 0.33 | -0.18 | 0.84 |
| Incredible Years (IY) | Conn (2018) | 13 | Total problems | CBCL | Parent/carer | Scale | 6 month follow-up | 4-6 months | 0.11 | -0.61 | 0.83 |
| Keeping foster and kinship carers supported (KEEP) | Chamberlain (2008) | 16 | Number of child problem behaviours/day | PDR | Parent/carer | Scale | 6 month follow-up | 4-6 months | -0.26 | -0.43 | -0.09 |
| Keeping foster and kinship carers supported (KEEP) | Price (2015) | 16 | Child behaviour problems | PDR | Parent/carer | Scale | Post test | 4-6 months | -0.01 | -0.22 | 0.21 |
| Life Story | Haight (2010) | 28 | Total problems | CBCL | Parent/carer | Scale | 12 month follow up | 7-12 months | -0.15 | -1.16 | 0.87 |
| Mentalization‐based therapy (MBT) | Midgley (2019) | 12 | Total problems | SDQ | Parent/carer | Scale | 24 week follow up | 4-6 months | 0.44 | 0.04 | 0.84 |
| Mentalization‐based therapy (MBT) | Midgley (2019) | 12 | Total problems | SDQ | Parent/carer | Scale | 12 week follow up | 0-3 months | 0.31 | -0.09 | 0.71 |
| Mentalization‐based therapy (MBT) | Midgley (2019) | 12 | Total problems | SDQ | Child/young person | Scale | 12 week follow up | 0-3 months | -0.67 | -1.12 | -0.22 |
| Mentalization‐based therapy (MBT) | Midgley (2019) | 12 | Total problems | SDQ | Child/young person | Scale | 24 week follow up | 4-6 months | -0.76 | -1.21 | -0.31 |
| Multidimensional Treatment Foster Care (MTFC) | Green (2014) | 52 | Nation Outcome Scales for Children and Adolescents | HoNOSCA | Multi informant | Scale | 12 month follow up | 7-12 months | -0.13 | -0.87 | 0.61 |
| Multidimensional Treatment Foster Care (MTFC) | Jonkman (2017) | 36 | Total problems | CBCL | Parent/carer | Scale | 12 month follow-up | 7-12 months | *Insufficient data to calculate effect size* | | |
| Multidimensional Treatment Foster Care (MTFC) | Jonkman (2017) | 36 | Total problems | TRF | Teacher | Scale | 12 month follow-up | 7-12 months | *Insufficient data to calculate effect size* | | |
| Nonviolent Resistance (NVR) Training | Van Holen (2018) | 10 | Total problems | CBCL | Parent/carer | Scale | 3 month follow up | 4-6 months | -0.33 | -0.83 | 0.17 |
| Nonviolent Resistance (NVR) Training | Van Holen (2018) | 10 | Total problems | CBCL | Parent/carer | Scale | Post intervention | 0-3 months | -0.36 | -0.87 | 0.14 |
| Parent Management Training Oregon (PMTO) | Akin (2018) | 24 | Problem behaviour | SSIS | Parent/carer | Scale | Six month post-test | 4-6 months | -0.15 | -0.34 | 0.03 |
| Parent Management Training Oregon (PMTO) | Akin (2018) | 24 | Problem behaviour | SSIS | Parent/carer | Scale | 12 month follow up | 7-12 months | -0.31 | -0.55 | -0.06 |
| Parent Management Training Oregon (PMTO) | Maaskant (2016) | 30 | Total problems | TRF | Teacher | Scale | 12 month follow up | 7-12 months | 0.37 | -0.12 | 0.86 |
| Parent Management Training Oregon (PMTO) | Maaskant (2016) | 30 | Total problems | CBCL | Parent/carer | Scale | 12 month follow up | 7-12 months | 0.00 | -0.35 | 0.35 |
| Parent Management Training Oregon (PMTO) | Maaskant (2016) | 30 | Total problems | CBCL | Parent/carer | Scale | 6 month follow-up | 4-6 months | -0.16 | -0.49 | 0.17 |
| Parent Management Training Oregon (PMTO) | Maaskant (2016) | 30 | Total problems | TRF | Teacher | Scale | 6 month follow-up | 4-6 months | -0.13 | -0.66 | 0.40 |
| Parent-child Interaction Therapy (PCIT) - Brief | Mersky (2016) | 8 | Problem behaviour | ECBI | Parent/carer | Scale | 14 week follow up | 4-6 months | -0.23 | -0.77 | 0.31 |
| Parent-child Interaction Therapy (PCIT) - Brief | Mersky (2016) | 8 | Problem behaviour | ECBI | Parent/carer | Scale | 8 week follow up | 0-3 months | -0.64 | -1.16 | -0.12 |
| Parent-child Interaction Therapy (PCIT) - Extended | Mersky (2016) | 8 | Problem behaviour | ECBI | Parent/carer | Scale | 8 week follow up | 0-3 months | -0.45 | -1.08 | 0.18 |
| Parent-child Interaction Therapy (PCIT) - Extended | Mersky (2016) | 8 | Problem behaviour | ECBI | Parent/carer | Scale | 14 week follow up | 4-6 months | -0.49 | -1.15 | 0.17 |
| Pathways Home | DeGarmo (2013) | 24 | Child problem behaviors | PDR | Parent/carer | Scale | 6 month follow-up | 4-6 months | -0.05 | -0.43 | 0.34 |

#### Total social, emotional and behavioural functioning and/or impaired functioning

| **Intervention** | **Lead author** | **Int. length (weeks)** | **Outcome assessed** | **Tool** | **Informant** | **Data type** | **Follow-up** | **Follow-up (category)** | **Effect size** | **95% CI (low)** | **95% CI (high)** |
| --- | --- | --- | --- | --- | --- | --- | --- | --- | --- | --- | --- |
| Adolescent HealthRHYTHMS Drumming Protocol - experimental | Bittman (2009) | 6 | Social adaptation | APS | Child/young person | Scale | 6 week follow up | 0-3 months | -0.13 | -0.79 | 0.53 |
| Adolescent HealthRHYTHMS Drumming Protocol - experimental | Bittman (2009) | 6 | Social-emotional functioning | CAFAS | Clinician | Scale | 6 week follow up | 0-3 months | -0.45 | -1.12 | 0.22 |
| Adolescent HealthRHYTHMS Drumming Protocol - extended | Bittman (2009) | 6 | Social adaptation | APS | Child/young person | Scale | 6 week follow up | 0-3 months | -0.11 | -0.77 | 0.55 |
| Adolescent HealthRHYTHMS Drumming Protocol - extended | Bittman (2009) | 6 | Social-emotional functioning | CAFAS | Clinician | Scale | 6 week follow up | 0-3 months | -0.62 | -1.30 | 0.06 |
| Adolescent HealthRHYTHMS Drumming Protocol - extended | Bittman (2009) | 6 | Interpersonal problems | APS | Child/young person | Scale | 6 week follow up | 0-3 months | -0.64 | -1.32 | 0.04 |
| Adolescent HealthRHYTHMS Drumming Protocol - extended | Bittman (2009) | 6 | Interpersonal problems | APS | Child/young person | Scale | 6 week follow up | 0-3 months | -0.89 | -1.59 | -0.20 |
| Cognitively-Based Compassion Training (CBCT) | Reddy (2013) | 6 | Emotional regulation | DERS | Unclear | Scale | Post intervention | 0-3 months | -0.09 | -0.55 | 0.38 |
| Connect-KP | Pasalich (2021) | 9 | Behavioural and emotional adjustment | SDQ | Parent/carer | Scale | 6 month follow up | 7-12 months | -0.44 | -1.24 | 0.35 |
| Connect-KP | Pasalich (2021) | 9 | Behavioural and emotional adjustment | SDQ | Parent/carer | Scale | Post-intervention | 0-3 months | -0.48 | -1.29 | 0.34 |
| Family Minds | Adkins (2021) | 5 | Peer Problems | SDQ | Parent/carer | Scale | Six week follow-up | 0-3 months | 0.34 | -0.21 | 0.89 |
| Family Minds | Adkins (2021) | 5 | Total difficulties | SDQ | Parent/carer | Scale | Six week follow-up | 0-3 months | 0.30 | -0.24 | 0.85 |
| Family Minds | Adkins (2021) | 5 | Prosocial behaviour | SDQ | Parent/carer | Scale | Six week follow-up | 0-3 months | 0.15 | -0.39 | 0.70 |
| Family Minds | Adkins (2021) | 5 | Emotional Symptoms | SDQ | Parent/carer | Scale | Six week follow-up | 0-3 months | 0.15 | -0.40 | 0.69 |
| Foster carer and foster children group-based intervention | Smith (2011) | 3 | Pro-social behavior | PDR | Parent/carer | Scale | Six month follow up | 4-6 months | -0.11 | -0.28 | 0.51 |
| Fostering Changes (FC) | Briskman (2012) | 12 | Hyperactivity | SDQ | Parent/carer | Scale | Post intervention | 0-3 months | 0.15 | -0.35 | 0.64 |
| Fostering Changes (FC) | Briskman (2012) | 12 | Peer relationships | SDQ | Parent/carer | Scale | Post intervention | 0-3 months | 0.05 | -0.45 | 0.54 |
| Fostering Changes (FC) | Briskman (2012) | 12 | Total problems | SDQ | Parent/carer | Scale | Post intervention | 0-3 months | -0.03 | -0.53 | 0.47 |
| Fostering Changes (FC) | Briskman (2012) | 12 | Pro-social behavior | SDQ | Parent/carer | Scale | Post intervention | 0-3 months | 0.04 | -0.53 | 0.46 |
| Fostering Changes (FC) | Briskman (2012) | 12 | Emotional Symptoms | SDQ | Parent/carer | Scale | Post intervention | 0-3 months | -0.08 | -0.58 | 0.41 |
| Fostering Changes (FC) | Briskman (2012) | 12 | Conduct problems | SDQ | Parent/carer | Scale | Post intervention | 0-3 months | -0.19 | -0.69 | 0.31 |
| Fostering Changes (FC) | Moody (2020) | 12 | Prosocial behaviour | SDQ | Parent/carer | Scale | 3 month follow up | 0-3 months | 0.12 | -0.15 | 0.39 |
| Fostering Changes (FC) | Moody (2020) | 12 | Prosocial behaviour | SDQ | Parent/carer | Scale | 12 month follow up | 7-12 months | 0.04 | -0.23 | 0.32 |
| Fostering Changes (FC) | Moody (2020) | 12 | Total difficulties | SDQ | Parent/carer | Scale | 12 month follow up | 7-12 months | -0.04 | -0.32 | 0.23 |
| Fostering Changes (FC) | Moody (2020) | 12 | Emotional problems | SDQ | Parent/carer | Scale | 12 month follow up | 7-12 months | -0.09 | -0.37 | 0.18 |
| Fostering Changes (FC) | Moody (2020) | 12 | Peer Problems | SDQ | Parent/carer | Scale | 12 month follow up | 7-12 months | -0.16 | -0.44 | 0.12 |
| Fostering Changes (FC) | Moody (2020) | 12 | Peer Problems | SDQ | Parent/carer | Scale | 3 month follow up | 0-3 months | -0.28 | -0.56 | -0.01 |
| Fostering Changes (FC) | Moody (2020) | 12 | Total difficulties | SDQ | Parent/carer | Scale | 3 month follow up | 0-3 months | -0.32 | -0.59 | -0.05 |
| Fostering Changes (FC) | Moody (2020) | 12 | Emotional problems | SDQ | Parent/carer | Scale | 3 month follow up | 0-3 months | -0.33 | -0.60 | -0.06 |
| Fostering Healthy Futures (FHF) | Taussig (2010) | 30 | Mental health functioning | Multi | Multi informant | Scale | Post intervention | 7-12 months | 0.07 | -0.25 | 0.39 |
| Fostering Healthy Futures (FHF) | Taussig (2010) | 30 | Mental health functioning | Multi | Multi informant | Scale | 6 month post-intervention | 13-18 months | -0.51 | -0.83 | -0.18 |
| Fostering Healthy Futures (FHF) | Taussig (2019) | 30 | Mental health functioning | Multi | Multi informant | Scale | 6 month post-intervention | 13-18 months | -0.25 | -0.46 | -0.04 |
| kConnect | Suomi (2020) | 36 | Total functioning | SDQ | Parent/carer | Scale | Post-test | 7-12 months | 0.24 | -0.11 | 0.60 |
| Mentoring intervention for teenage pregnancy | Mezey (2015) | 52 | Emotional health - OK or better | Not stated | Child/young person | Categorical | 1 year | 7-12 months | -0.19 | -0.54 | 0.92 |
| Multidimensional Treatment Foster Care (MTFC) | Biehal (2012) | 36 | Child global functioning | CGAS | Multi informant | Scale | 12 month follow up | 7-12 months | -0.12 | -0.62 | 0.86 |
| Multidimensional Treatment Foster Care (MTFC) | Biehal (2012) | 36 | Child MH symptoms and social/physical functioning | HoNOSCA | Multi informant | Scale | 12 month follow up | 7-12 months | -0.16 | -0.90 | 0.58 |
| Multidimensional Treatment Foster Care (MTFC) | Green (2014) | 52 | Children's Global Assessment Scale | CGAS | Multi informant | Scale | 12 month follow up | 7-12 months | -0.07 | -0.67 | 0.81 |
| Parent Management Training Oregon (PMTO) | Akin (2018) | 24 | Social-emotional functioning | CAFAS | Parent/carer | Scale | Six month post-test | 4-6 months | -0.19 | -0.35 | -0.04 |
| Parent Management Training Oregon (PMTO) | Akin (2018) | 24 | Social-emotional functioning | CAFAS | Parent/carer | Scale | 12 month follow up | 7-12 months | -0.38 | -0.57 | -0.20 |
| Parent-Child Interaction Therapy (PCIT) - Brief + Extended | Mersky (2020) | 8 | Lability / negativity | ERC | Parent/carer | Scale | Post-test | 0-3 months | -0.67 | -1.15 | -0.19 |
| Supporting Looked After Children and Care Leavers In Decreasing Drugs (SOLID) | Alderson (2020) | 12 | Emotional problems | SDQ | Child/young person | Scale | 12 month follow-up | 7-12 months | *Insufficient data to calculate effect size* | | |
| Supporting Looked After Children and Care Leavers In Decreasing Drugs (SOLID) | Alderson (2020) | 12 | Peer problems | SDQ | Child/young person | Scale | 12 month follow-up | 7-12 months | *Insufficient data to calculate effect size* | | |
| Supporting Looked After Children and Care Leavers In Decreasing Drugs (SOLID) | Alderson (2020) | 12 | Prosocial behaviour | SDQ | Child/young person | Scale | 12 month follow-up | 7-12 months | *Insufficient data to calculate effect size* | | |
| Supporting Looked After Children and Care Leavers In Decreasing Drugs (SOLID) | Alderson (2020) | 12 | Total difficulties | SDQ | Child/young person | Scale | 12 month follow-up | 7-12 months | *Insufficient data to calculate effect size* | | |
| Wave by Wave Surf Therapy | Pereira (2020) | 21 | Behavioural and emotional problems | SDQ | Child/young person | Scale | Post-test | 4-6 months | 0.13 | -0.38 | 0.63 |
| Wave by Wave Surf Therapy | Pereira (2020) | 21 | Behavioural and emotional problems | SDQ | Parent/carer | Scale | Post-test | 4-6 months | 0.01 | -0.50 | 0.51 |
| Wave by Wave Surf Therapy | Pereira (2020) | 21 | Emotional regulation | ERQ-CA | Child/young person | Scale | Post-test | 4-6 months | -0.07 | -0.58 | 0.43 |

#### Internalizing behaviour problems

| **Intervention** | **Study design** | **Int. length (weeks)** | **Outcome assessed** | **Tool** | **Informant** | **Data type** | **Follow-up** | **Follow-up (category)** | **Effect size** | **95% CI (low)** | **95% CI (high)** |
| --- | --- | --- | --- | --- | --- | --- | --- | --- | --- | --- | --- |
| Adolescent HealthRHYTHMS Drumming Protocol - experimental | Bittman (2009) | 6 | Somatic complaints | RADS2 | Child/young person | Scale | 6 week follow up | 0-3 months | -0.38 | -1.05 | 0.29 |
| Adolescent HealthRHYTHMS Drumming Protocol - extended | Bittman (2009) | 6 | Somatic complaints | RADS2 | Child/young person | Scale | 6 week follow up | 0-3 months | -0.54 | -1.21 | 0.14 |
| **Attachment and Biobehavioural Catchup (ABC)** | Sprang (2009) | 10 | **Internalizing behaviour** | **CBCL** | **Parent/carer** | **Scale** | **Post intervention** | **0-3 months** | **-1.60** | **-2.22** | **-0.98** |
| Child Directed Interaction Training (CDIT) | N'Zi (2016) | 4 | Internalizing behaviour | CBCL | Parent/carer | Scale | 7 week follow up | 0-3 months | -0.22 | -1.24 | 0.80 |
| Cognitive and Affective Bibliotherapy - Affective | Betzalel (2010) | 8 | Psychosomatic complaints | TRF | Clinician | Scale | Follow-up | 4-6 months | 0.00 | -0.54 | 0.54 |
| **Cognitive and Affective Bibliotherapy - Affective** | Betzalel (2010) | 8 | **Withdrawal** | **TRF** | **Clinician** | **Scale** | **Follow-up** | **4-6 months** | **-0.75** | **-1.31** | **-0.18** |
| **Cognitive and Affective Bibliotherapy - Affective** | Betzalel (2010) | 8 | **Internalizing beahvior** | **TRF** | **Clinician** | **Scale** | **Follow-up** | **4-6 months** | **-0.77** | **-1.33** | **-0.21** |
| Cognitive and Affective Bibliotherapy - Cognitive | Betzalel (2010) | 8 | Withdrawal | TRF | Clinician | Scale | Follow-up | 4-6 months | 0.10 | -0.44 | 0.64 |
| Cognitive and Affective Bibliotherapy - Cognitive | Betzalel (2010) | 8 | Internalizing beahvior | TRF | Clinician | Scale | Follow-up | 4-6 months | -0.09 | -0.63 | 0.45 |
| Cognitive and Affective Bibliotherapy - Cognitive | Betzalel (2010) | 8 | Psychosomatic complaints | TRF | Clinician | Scale | Follow-up | 4-6 months | -0.34 | -0.88 | 0.20 |
| **Family Finding** | Vandivere (2017) | 20 | **Internalizing - clinical level** | **YSR** | **Child/young person** | **Categorical** | **24 month follow up** | **19-24 months** | **0.26** | **0.10** | **0.42** |
| Family Finding | Vandivere (2017) | 20 | Internalizing - borderline or clinical level | YSR | Child/young person | Categorical | 12 month follow up | 7-12 months | 0.14 | -0.15 | 0.44 |
| Family Finding | Vandivere (2017) | 20 | Internalizing - clinical level | YSR | Child/young person | Categorical | 12 month follow up | 7-12 months | 0.20 | -0.19 | 0.60 |
| Family Finding | Vandivere (2017) | 20 | Internalizing - borderline or clinical level | YSR | Child/young person | Categorical | 24 month follow up | 19-24 months | 0.18 | -0.28 | 0.64 |
| **Foster carer and foster children group-based intervention** | Smith (2011) | 3 | **Internalizing beahvior** | **PDR** | **Parent/carer** | **Scale** | **Six month follow up** | **4-6 months** | **-0.29** | **-0.51** | **-0.07** |
| Foster Parent Intervention | Van Holen (2017) | 10 | Internalaizing problems | CBCL | Parent/carer | Scale | Post intervention | 0-3 months | -0.45 | -1.91 | 1.01 |
| Foster Parent Intervention | Van Holen (2017) | 10 | Internalaizing problems | CBCL | Parent/carer | Scale | 3 month follow up | 4-6 months | -0.88 | -2.62 | 0.86 |
| Fostering Individualised Assistance Program (FIAP) | Clark (1994) | 72 | Internalizing beahvior | CBCL | Parent/carer | Scale | 18 month follow-up | 13-18 months | -0.01 | -0.39 | 0.37 |
| Fostering Individualised Assistance Program (FIAP) | Clark (1994) | 72 | Somatic complaints | YSR | Child/young person | Scale | 18 month follow-up | 13-18 months | -0.15 | -0.54 | 0.23 |
| Fostering Individualised Assistance Program (FIAP) | Clark (1994) | 72 | Somatic | CBCL | Parent/carer | Scale | 18 month follow-up | 13-18 months | -0.17 | -0.55 | 0.21 |
| Fostering Individualised Assistance Program (FIAP) | Clark (1994) | 72 | Withdrawn | YSR | Child/young person | Scale | 18 month follow-up | 13-18 months | -0.20 | -0.58 | 0.18 |
| Fostering Individualised Assistance Program (FIAP) | Clark (1994) | 72 | Internalizing beahvior | YSR | Child/young person | Scale | 18 month follow-up | 13-18 months | -0.23 | -0.61 | 0.16 |
| Incredible Years (IY) | Conn (2018) | 13 | Internalizing beahvior | CBCL | Parent/carer | Scale | 6 month follow-up | 4-6 months | -0.07 | -0.79 | 0.65 |
| kConnect | Suomi (2020) | 36 | Internalizing problems | SDQ | Parent/carer | Scale | Post-test | 7-12 months | 0.16 | -0.20 | 0.51 |
| Keeping foster and kinship carers supported (KEEP) | Price (2019) | 16 | Somatic complaints | CBCL | Parent/carer | Scale | Post-test | 4-6 months | -0.08 | -0.30 | 0.14 |
| Keeping foster and kinship carers supported (KEEP) | Price (2019) | 16 | Internalizing beahvior | CBCL | Parent/carer | Scale | Post-test | 4-6 months | -0.11 | -0.34 | 0.11 |
| Keeping foster and kinship carers supported (KEEP) | Price (2019) | 16 | Somatic complaints - above clinical cutoff | CBCL | Parent/carer | Categorical | Post-test | 4-6 months | -0.16 | -0.43 | 0.12 |
| **Keeping foster and kinship carers supported (KEEP)** | Price (2019) | 16 | **Internalizing beahvior - above borderline cutoff** | **CBCL** | **Parent/carer** | **Categorical** | **Post-test** | **4-6 months** | **-0.23** | **-0.45** | **-0.01** |
| **Keeping foster and kinship carers supported (KEEP)** | Price (2019) | 16 | **Internalizing beahvior - above clinical cutoff** | **CBCL** | **Parent/carer** | **Categorical** | **Post-test** | **4-6 months** | **-0.23** | **-0.45** | **-0.01** |
| Keeping foster and kinship carers supported (KEEP) | Price (2019) | 16 | Somatic complaints - above borderline cutoff | CBCL | Parent/carer | Categorical | Post-test | 4-6 months | -0.53 | -1.09 | 0.03 |
| Life Story | Haight (2010) | 28 | Internalizing beahvior | CBCL | Parent/carer | Scale | 12 month follow up | 7-12 months | 0.33 | -0.69 | 1.35 |
| **Mentalization‐based therapy (MBT)** | Midgley (2019) | 12 | **Internalizing beahvior** | **SDQ** | **Parent/carer** | **Scale** | **24 week follow up** | **4-6 months** | **0.48** | **0.13** | **0.83** |
| **Mentalization‐based therapy (MBT)** | Midgley (2019) | 12 | **Internalizing beahvior** | **SDQ** | **Parent/carer** | **Scale** | **12 week follow up** | **0-3 months** | **0.35** | **0.00** | **0.70** |
| **Mentalization‐based therapy (MBT)** | Midgley (2019) | 12 | **Internalizing beahvior** | **SDQ** | **Child/young person** | **Scale** | **12 week follow up** | **0-3 months** | **-1.04** | **-1.54** | **-0.54** |
| **Mentalization‐based therapy (MBT)** | Midgley (2019) | 12 | **Internalizing beahvior** | **SDQ** | **Child/young person** | **Scale** | **24 week follow up** | **4-6 months** | **-1.30** | **-1.80** | **-0.80** |
| Multidimensional Treatment Foster Care (MTFC) | Jonkman (2017) | 36 | Internalizing problems | CBCL | Parent/carer | Scale | 12 month follow-up | 7-12 months | *Insufficient data to calculate effect size* | | |
| Multidimensional Treatment Foster Care (MTFC) | Jonkman (2017) | 36 | Internalizing problems | TRF | Teacher | Scale | 12 month follow-up | 7-12 months | *Insufficient data to calculate effect size* | | |
| Nonviolent Resistance (NVR) Training | Van Holen (2018) | 10 | Internalaizing problems | CBCL | Parent/carer | Scale | Post intervention | 0-3 months | -0.17 | -0.67 | 0.33 |
| Nonviolent Resistance (NVR) Training | Van Holen (2018) | 10 | Internalaizing problems | CBCL | Parent/carer | Scale | 3 month follow up | 4-6 months | -0.21 | -0.71 | 0.29 |
| Parent Management Training Oregon (PMTO) | Maaskant (2016) | 30 | Internalizing problems | TRF | Teacher | Scale | 12 month follow up | 7-12 months | 0.30 | -0.17 | 0.77 |
| Parent Management Training Oregon (PMTO) | Maaskant (2016) | 30 | Internalizing problems | CBCL | Parent/carer | Scale | 6 month follow-up | 4-6 months | 0.14 | -0.25 | 0.53 |
| Parent Management Training Oregon (PMTO) | Maaskant (2016) | 30 | Internalizing problems | CBCL | Parent/carer | Scale | 12 month follow up | 7-12 months | 0.12 | -0.27 | 0.51 |
| Parent Management Training Oregon (PMTO) | Maaskant (2016) | 30 | Internalizing problems | TRF | Teacher | Scale | 6 month follow-up | 4-6 months | -0.03 | -0.48 | 0.42 |
| Parent-child Interaction Therapy (PCIT) - Brief | Mersky (2016) | 8 | Internalising problems | CBCL | Parent/carer | Scale | 14 week follow up | 4-6 months | -0.31 | -0.85 | 0.23 |
| Parent-child Interaction Therapy (PCIT) - Brief | Mersky (2016) | 8 | Internalising problems | CBCL | Parent/carer | Scale | 8 week follow up | 0-3 months | -0.44 | -0.95 | 0.08 |
| Parent-child Interaction Therapy (PCIT) - Extended | Mersky (2016) | 8 | Internalising problems | CBCL | Parent/carer | Scale | 8 week follow up | 0-3 months | 0.34 | -0.29 | 0.97 |
| Parent-child Interaction Therapy (PCIT) - Extended | Mersky (2016) | 8 | Internalising problems | CBCL | Parent/carer | Scale | 14 week follow up | 4-6 months | -0.28 | -0.93 | 0.38 |
| Take Charge | Geenen (2012) | 20 | Somatic complaints | CBCL | Parent/carer | Scale | 12 month follow up | 7-12 months | -0.30 | -0.65 | 0.06 |
| **Take Charge** | Geenen (2012) | 20 | **Somatic complaints** | **CBCL** | **Parent/carer** | **Scale** | **18 month follow-up** | **13-18 months** | **-0.54** | **-0.90** | **-0.18** |
| Teach Your Children Well | Marquis (2014) | 30 | Internalizing beahvior | CBCL | Parent/carer | Scale | 9 month follow up | 7-12 months | -0.30 | -0.80 | 0.19 |

#### Externalizing behaviour problems

| **Intervention** | **Lead author** | **Int. length (weeks)** | **Outcome assessed** | **Tool** | **Informant** | **Data type** | **Follow-up** | **Follow-up (category)** | **Effect size** | **95% CI (low)** | **95% CI (high)** |
| --- | --- | --- | --- | --- | --- | --- | --- | --- | --- | --- | --- |
| Adolescent HealthRHYTHMS Drumming Protocol - experimental | Bittman (2009) | 6 | Aggression | APS | Child/young person | Scale | 6 week follow up | 0-3 months | -0.27 | -0.94 | 0.39 |
| Adolescent HealthRHYTHMS Drumming Protocol - experimental | Bittman (2009) | 6 | Anger | APS | Child/young person | Scale | 6 week follow up | 0-3 months | -0.53 | -1.20 | 0.15 |
| Adolescent HealthRHYTHMS Drumming Protocol - extended | Bittman (2009) | 6 | Anger (total) | AARS | Child/young person | Scale | 6 week follow up | 0-3 months | -0.14 | -0.81 | 0.52 |
| Adolescent HealthRHYTHMS Drumming Protocol - extended | Bittman (2009) | 6 | Aggression | APS | Child/young person | Scale | 6 week follow up | 0-3 months | -0.40 | -1.07 | 0.27 |
| Adolescent HealthRHYTHMS Drumming Protocol - extended | Bittman (2009) | 6 | Anger (total) | AARS | Child/young person | Scale | 6 week follow up | 0-3 months | -0.50 | -1.18 | 0.17 |
| Adolescent HealthRHYTHMS Drumming Protocol - extended | Bittman (2009) | 6 | Anger | APS | Child/young person | Scale | 6 week follow up | 0-3 months | -0.94 | -1.64 | -0.24 |
| Attachment and Biobehavioural Catchup (ABC) | Sprang (2009) | 10 | Externalizing behaviour | CBCL | Parent/carer | Scale | Post intervention | 0-3 months | -1.80 | -2.44 | -1.16 |
| Child Adult Relationship Enhancement (CARE) | Messer (2018) | 4 | Anger / aggression | TSCYC | Parent/carer | Scale | 1 monh follow up | 0-3 months | -0.35 | -1.59 | 0.89 |
| Child Adult Relationship Enhancement (CARE) | Messer (2018) | 4 | Anger / aggression | TSCYC | Parent/carer | Scale | 3 month follow up | 4-6 months | -0.96 | -2.38 | 0.46 |
| Child Directed Interaction Training (CDIT) | N'Zi (2016) | 4 | Externalizing behaviour | CBCL | Parent/carer | Scale | 7 week follow up | 0-3 months | -1.05 | -2.13 | 0.04 |
| Cognitive and Affective Bibliotherapy - Affective | Betzalel (2010) | 8 | Delinquency | TRF | Clinician | Scale | Follow-up | 4-6 months | -0.96 | -1.54 | -0.39 |
| Cognitive and Affective Bibliotherapy - Affective | Betzalel (2010) | 8 | Aggression | TRF | Clinician | Scale | Follow-up | 4-6 months | -0.99 | -1.57 | -0.42 |
| Cognitive and Affective Bibliotherapy - Affective | Betzalel (2010) | 8 | Externalizing behaviour | TRF | Clinician | Scale | Follow-up | 4-6 months | -1.07 | -1.65 | -0.49 |
| Cognitive and Affective Bibliotherapy - Cognitive | Betzalel (2010) | 8 | Aggression | TRF | Clinician | Scale | Follow-up | 4-6 months | -0.46 | -1.00 | 0.09 |
| Cognitive and Affective Bibliotherapy - Cognitive | Betzalel (2010) | 8 | Delinquency | TRF | Clinician | Scale | Follow-up | 4-6 months | -0.47 | -1.01 | 0.08 |
| Cognitive and Affective Bibliotherapy - Cognitive | Betzalel (2010) | 8 | Externalizing behaviour | TRF | Clinician | Scale | Follow-up | 4-6 months | -0.50 | -1.04 | 0.05 |
| Connect-KP | Pasalich (2021) | 9 | Affect dyscontrol | ARC | Parent/carer | Scale | 6 month follow up | 7-12 months | -0.60 | -1.40 | 0.20 |
| Connect-KP | Pasalich (2021) | 9 | Affect dyscontrol | ARC | Parent/carer | Scale | Post-intervention | 0-3 months | -0.97 | -1.81 | -0.12 |
| Dojo: Biofeedback videogame | Schuurmans (2018) | 8 | Externalizing problems | SDQ | Parent/carer | Scale | Post intervention | 0-3 months | -0.09 | -0.74 | 0.57 |
| Dojo: Biofeedback videogame | Schuurmans (2018) | 8 | Externalizing problems | SDQ | Parent/carer | Scale | Follow-up | 4-6 months | -0.20 | -0.96 | 0.56 |
| Dojo: Biofeedback videogame | Schuurmans (2018) | 8 | Externalizing problems | SDQ | Child/young person | Scale | Post intervention | 0-3 months | -0.85 | -1.53 | -0.17 |
| Dojo: Biofeedback videogame | Schuurmans (2018) | 8 | Externalizing problems | SDQ | Child/young person | Scale | Follow-up | 4-6 months | -1.16 | -1.87 | -0.45 |
| Family Finding | Vandivere (2017) | 20 | Externalizing - clinical level | YSR | Child/young person | Categorical | 24 month follow up | 19-24 months | 0.19 | 0.03 | 0.36 |
| Family Finding | Vandivere (2017) | 20 | Externalizing - clinical level | YSR | Child/young person | Categorical | 12 month follow up | 7-12 months | 0.35 | 0.02 | 0.68 |
| Family Finding | Vandivere (2017) | 20 | Externalizing - borderline or clinical level | YSR | Child/young person | Categorical | 12 month follow up | 7-12 months | 0.07 | -0.21 | 0.35 |
| Family Finding | Vandivere (2017) | 20 | Externalizing - borderline or clinical level | YSR | Child/young person | Categorical | 24 month follow up | 19-24 months | 0.17 | -0.64 | 0.97 |
| Family Minds | Adkins (2021) | 5 | Conduct problems | SDQ | Parent/carer | Scale | Six week follow-up | 0-3 months | 0.11 | -0.44 | 0.65 |
| Foster carer and foster children group-based intervention | Smith (2011) | 3 | Externalizing behaviour | PDR | Parent/carer | Scale | Six month follow up | 4-6 months | -0.09 | -0.16 | -0.02 |
| Foster Parent Intervention | Van Holen (2017) | 10 | Externalizing problems | CBCL | Parent/carer | Scale | Post intervention | 0-3 months | -0.26 | -2.25 | 1.73 |
| Foster Parent Intervention | Van Holen (2017) | 10 | Externalizing problems | CBCL | Parent/carer | Scale | 3 month follow up | 4-6 months | -0.84 | -2.60 | 0.93 |
| Fostering Changes (FC) | Moody (2020) | 12 | Conduct problems | SDQ | Parent/carer | Scale | 12 month follow up | 7-12 months | 0.15 | -0.12 | 0.43 |
| Fostering Changes (FC) | Moody (2020) | 12 | Conduct problems | SDQ | Parent/carer | Scale | 3 month follow up | 0-3 months | -0.08 | -0.35 | 0.19 |
| Fostering Individualised Assistance Program (FIAP) | Clark (1994) | 72 | Delinquency | CBCL | Parent/carer | Scale | 18 month follow-up | 13-18 months | -0.06 | -0.44 | 0.32 |
| Fostering Individualised Assistance Program (FIAP) | Clark (1994) | 72 | Externalizing behaviour | CBCL | Parent/carer | Scale | 18 month follow-up | 13-18 months | -0.20 | -0.58 | 0.18 |
| Fostering Individualised Assistance Program (FIAP) | Clark (1994) | 72 | Aggression | CBCL | Parent/carer | Scale | 18 month follow-up | 13-18 months | -0.24 | -0.62 | 0.14 |
| Fostering Individualised Assistance Program (FIAP) | Clark (1994) | 72 | Delinquency | YSR | Child/young person | Scale | 18 month follow-up | 13-18 months | -0.33 | -0.72 | 0.05 |
| Fostering Individualised Assistance Program (FIAP) | Clark (1994) | 72 | Aggression | YSR | Child/young person | Scale | 18 month follow-up | 13-18 months | -0.42 | -0.80 | -0.03 |
| Fostering Individualised Assistance Program (FIAP) | Clark (1994) | 72 | Externalizing behaviour | YSR | Child/young person | Scale | 18 month follow-up | 13-18 months | -0.43 | -0.82 | -0.05 |
| Head Start | Lipscomb (2013) | 52 | Externalizing behaviour | ASPI | Teacher | Scale | 12 month follow-up | 7-12 months | *Insufficient data to calculate effect size* | | |
| Incredible Years (IY) | Conn (2018) | 13 | Externalizing behaviour | CBCL | Parent/carer | Scale | 6 month follow-up | 4-6 months | 0.18 | -0.54 | 0.90 |
| Incredible Years (IY) | Linares (2006) | 12 | Disruptive classroom behaviour | SESBI-R | Teacher | Scale | Six month post-test | 4-6 months | 0.32 | -0.10 | 0.74 |
| Incredible Years (IY) | Linares (2006) | 12 | Disruptive classroom behaviour | SESBI-R | Teacher | Scale | Post intervention | 0-3 months | 0.05 | -0.34 | 0.44 |
| Incredible Years (IY) | Linares (2006) | 12 | Externalizing behaviour | CBCL | Parent/carer | Scale | Post intervention | 0-3 months | -0.14 | -0.54 | 0.26 |
| Incredible Years (IY) | Linares (2006) | 12 | Externalizing problems | ECBI | Parent/carer | Scale | Post intervention | 0-3 months | -0.23 | -0.63 | 0.17 |
| Incredible Years (IY) | Linares (2006) | 12 | Externalizing problems | ECBI | Parent/carer | Scale | Six month post-test | 4-6 months | -0.33 | -0.75 | 0.09 |
| Incredible Years (IY) | Linares (2006) | 12 | Externalizing behaviour | CBCL | Parent/carer | Scale | Six month post-test | 4-6 months | -0.36 | -0.78 | 0.06 |
| kConnect | Suomi (2020) | 36 | Externalizing problems | SDQ | Parent/carer | Scale | Post-test | 7-12 months | 0.25 | -0.11 | 0.60 |
| Keeping foster and kinship carers supported (KEEP) | Price (2019) | 16 | Rule breaking | CBCL | Parent/carer | Scale | Post-test | 4-6 months | 0.05 | -0.17 | 0.27 |
| Keeping foster and kinship carers supported (KEEP) | Price (2019) | 16 | Externalizing behaviour  - above borderline cutoff | CBCL | Parent/carer | Categorical | Post-test | 4-6 months | -0.01 | -0.23 | 0.22 |
| Keeping foster and kinship carers supported (KEEP) | Price (2019) | 16 | Aggression | CBCL | Parent/carer | Scale | Post-test | 4-6 months | -0.01 | -0.24 | 0.21 |
| Keeping foster and kinship carers supported (KEEP) | Price (2019) | 16 | Aggression - above borderline cutoff | CBCL | Parent/carer | Categorical | Post-test | 4-6 months | -0.02 | -0.24 | 0.21 |
| Keeping foster and kinship carers supported (KEEP) | Price (2019) | 16 | Aggression - above clinical cutoff | CBCL | Parent/carer | Categorical | Post-test | 4-6 months | -0.04 | -0.25 | 0.17 |
| Keeping foster and kinship carers supported (KEEP) | Price (2019) | 16 | Externalizing behaviour | CBCL | Parent/carer | Scale | Post-test | 4-6 months | -0.05 | -0.28 | 0.17 |
| Keeping foster and kinship carers supported (KEEP) | Price (2019) | 16 | Rule breaking - above clinical cutoff | CBCL | Parent/carer | Categorical | Post-test | 4-6 months | -0.11 | -0.49 | 0.26 |
| Keeping foster and kinship carers supported (KEEP) | Price (2019) | 16 | Externalizing behaviour  - above clinical cutoff | CBCL | Parent/carer | Categorical | Post-test | 4-6 months | -0.14 | -0.51 | 0.24 |
| Keeping foster and kinship carers supported (KEEP) | Price (2019) | 16 | Rule breaking - above borderline cutoff | CBCL | Parent/carer | Categorical | Post-test | 4-6 months | -0.29 | -0.57 | -0.01 |
| Life Story | Haight (2010) | 28 | Externalizing behaviour | CBCL | Parent/carer | Scale | 12 month follow up | 7-12 months | -0.57 | -1.61 | 0.46 |
| Mentalization‐based therapy (MBT) | Midgley (2019) | 12 | Externalizing problems | SDQ | Parent/carer | Scale | 24 week follow up | 4-6 months | 0.20 | -0.15 | 0.55 |
| Mentalization‐based therapy (MBT) | Midgley (2019) | 12 | Externalizing problems | SDQ | Parent/carer | Scale | 12 week follow up | 0-3 months | 0.04 | -0.31 | 0.39 |
| Mentalization‐based therapy (MBT) | Midgley (2019) | 12 | Externalizing problems | SDQ | Child/young person | Scale | 24 week follow up | 4-6 months | -0.09 | -0.59 | 0.41 |
| Mentalization‐based therapy (MBT) | Midgley (2019) | 12 | Externalizing problems | SDQ | Child/young person | Scale | 12 week follow up | 0-3 months | -0.15 | -0.60 | 0.30 |
| Multidimensional Treatment Foster Care (MTFC) | Jonkman (2017) | 36 | Externalizing problems | CBCL | Parent/carer | Scale | 12 month follow-up | 7-12 months | *Insufficient data to calculate effect size* | | |
| Multidimensional Treatment Foster Care (MTFC) | Jonkman (2017) | 36 | Externalizing problems | TRF | Teacher | Scale | 12 month follow-up | 7-12 months | *Insufficient data to calculate effect size* | | |
| Nonviolent Resistance (NVR) Training | Van Holen (2018) | 10 | Externalizing problems | CBCL | Parent/carer | Scale | Post intervention | 0-3 months | -0.13 | -0.63 | 0.37 |
| Nonviolent Resistance (NVR) Training | Van Holen (2018) | 10 | Externalizing problems | CBCL | Parent/carer | Scale | 3 month follow up | 4-6 months | -0.21 | -0.71 | 0.29 |
| Parent Management Training Oregon (PMTO) | Maaskant (2016) | 30 | Externalizing problems | TRF | Teacher | Scale | 12 month follow up | 7-12 months | 0.08 | -0.10 | 0.26 |
| Parent Management Training Oregon (PMTO) | Maaskant (2016) | 30 | Externalizing problems | CBCL | Parent/carer | Scale | 12 month follow up | 7-12 months | -0.12 | -0.49 | 0.25 |
| Parent Management Training Oregon (PMTO) | Maaskant (2016) | 30 | Externalizing problems | TRF | Teacher | Scale | 6 month follow-up | 4-6 months | -0.04 | -0.53 | 0.45 |
| Parent Management Training Oregon (PMTO) | Maaskant (2016) | 30 | Externalizing problems | CBCL | Parent/carer | Scale | 6 month follow-up | 4-6 months | -0.33 | -0.66 | 0.00 |
| Parent-child Interaction Therapy (PCIT) - Brief | Mersky (2016) | 8 | Externalising problems | CBCL | Parent/carer | Scale | 14 week follow up | 4-6 months | -0.25 | -0.79 | 0.29 |
| Parent-child Interaction Therapy (PCIT) - Brief | Mersky (2016) | 8 | Externalising problems | CBCL | Parent/carer | Scale | 8 week follow up | 0-3 months | -0.49 | -1.00 | 0.03 |
| Parent-child Interaction Therapy (PCIT) - Extended | Mersky (2016) | 8 | Externalising problems | CBCL | Parent/carer | Scale | 8 week follow up | 0-3 months | -0.22 | -0.84 | 0.41 |
| Parent-child Interaction Therapy (PCIT) - Extended | Mersky (2016) | 8 | Externalising problems | CBCL | Parent/carer | Scale | 14 week follow up | 4-6 months | -0.57 | -1.24 | 0.10 |
| Supporting Looked After Children and Care Leavers In Decreasing Drugs (SOLID) | Alderson (2020) | 12 | Conduct problems | SDQ | Child/young person | Scale | 12 month follow-up | 7-12 months | *Insufficient data to calculate effect size* | | |
| Supporting Looked After Children and Care Leavers In Decreasing Drugs (SOLID) | Alderson (2020) | 12 | Hyperactivity | SDQ | Child/young person | Scale | 12 month follow-up | 7-12 months | *Insufficient data to calculate effect size* | | |
| Teach Your Children Well | Marquis (2014) | 30 | Externalzing behaviour | CBCL | Parent/carer | Scale | 9 month follow up | 7-12 months | -0.13 | -0.62 | 0.36 |
| Triple P for Foster Parents | Job (2022) | 5 | Externalizing - intensity scale | ECBI | Parent/carer | Scale | 12 month follow-up (m = 13 months) | 13-18 months | 0.33 | -0.12 | 0.79 |
| Triple P for Foster Parents | Job (2022) | 5 | Externalizing - intensity scale | ECBI | Parent/carer | Scale | 6 month follow-up (m = 7 months) | 7-12 months | 0.10 | -0.36 | 0.55 |

#### Depression and anxiety

| **Intervention** | **Lead author** | **Int. length (weeks)** | **Outcome assessed** | **Tool** | **Informant** | **Data type** | **Follow-up** | **Follow-up (category)** | **Effect size** | **95% CI (low)** | **95% CI (high)** |
| --- | --- | --- | --- | --- | --- | --- | --- | --- | --- | --- | --- |
| Adolescent HealthRHYTHMS Drumming Protocol - experimental | Bittman (2009) | 6 | Depression | RADS2 | Child/young person | Scale | 6 week follow up | 0-3 months | -0.57 | -1.24 | 0.11 |
| Adolescent HealthRHYTHMS Drumming Protocol - extended | Bittman (2009) | 6 | Depression | RADS2 | Child/young person | Scale | 6 week follow up | 0-3 months | -0.65 | -1.33 | 0.03 |
| Child Adult Relationship Enhancement (CARE) | Messer (2018) | 4 | Anxiety | TSCYC | Parent/carer | Scale | 1 monh follow up | 0-3 months | -1.00 | -2.27 | 0.26 |
| Child Adult Relationship Enhancement (CARE) | Messer (2018) | 4 | Depression | TSCYC | Parent/carer | Scale | 1 monh follow up | 0-3 months | -1.03 | -2.29 | 0.24 |
| Child Adult Relationship Enhancement (CARE) | Messer (2018) | 4 | Depression | TSCYC | Parent/carer | Scale | 3 month follow up | 4-6 months | -1.27 | -2.71 | 0.17 |
| Child Adult Relationship Enhancement (CARE) | Messer (2018) | 4 | Anxiety | TSCYC | Parent/carer | Scale | 3 month follow up | 4-6 months | -1.59 | -3.04 | -0.14 |
| Cognitive and Affective Bibliotherapy - Affective | Betzalel (2010) | 8 | Physiological (anxiety) | RCMAS | Child/young person | Scale | Post intervention | 0-3 months | 0.10 | -0.45 | 0.64 |
| Cognitive and Affective Bibliotherapy - Affective | Betzalel (2010) | 8 | Physiological (anxiety) | RCMAS | Child/young person | Scale | Follow-up | 4-6 months | 0.03 | -0.51 | 0.58 |
| Cognitive and Affective Bibliotherapy - Affective | Betzalel (2010) | 8 | Worry (anxiety) | RCMAS | Child/young person | Scale | Post intervention | 0-3 months | 0.00 | -0.55 | 0.54 |
| Cognitive and Affective Bibliotherapy - Affective | Betzalel (2010) | 8 | Anxiety total | RCMAS | Child/young person | Scale | Post intervention | 0-3 months | 0.00 | -0.55 | 0.54 |
| Cognitive and Affective Bibliotherapy - Affective | Betzalel (2010) | 8 | Social (anxiety) | RCMAS | Child/young person | Scale | Follow-up | 4-6 months | -0.10 | -0.64 | 0.44 |
| Cognitive and Affective Bibliotherapy - Affective | Betzalel (2010) | 8 | Social (anxiety) | RCMAS | Child/young person | Scale | Post intervention | 0-3 months | -0.13 | -0.67 | 0.42 |
| Cognitive and Affective Bibliotherapy - Affective | Betzalel (2010) | 8 | Anxiety total | RCMAS | Child/young person | Scale | Follow-up | 4-6 months | -0.20 | -0.74 | 0.35 |
| Cognitive and Affective Bibliotherapy - Affective | Betzalel (2010) | 8 | Worry (anxiety) | RCMAS | Child/young person | Scale | Follow-up | 4-6 months | -0.40 | -0.95 | 0.15 |
| Cognitive and Affective Bibliotherapy - Affective | Betzalel (2010) | 8 | Anxiety/depression | TRF | Clinician | Scale | Follow-up | 4-6 months | -0.73 | -1.29 | -0.17 |
| Cognitive and Affective Bibliotherapy - Cognitive | Betzalel (2010) | 8 | Social (anxiety) | RCMAS | Child/young person | Scale | Post intervention | 0-3 months | -0.03 | -0.56 | 0.51 |
| Cognitive and Affective Bibliotherapy - Cognitive | Betzalel (2010) | 8 | Social (anxiety) | RCMAS | Child/young person | Scale | Follow-up | 4-6 months | -0.06 | -0.60 | 0.48 |
| Cognitive and Affective Bibliotherapy - Cognitive | Betzalel (2010) | 8 | Anxiety/depression | TRF | Clinician | Scale | Follow-up | 4-6 months | -0.07 | -0.61 | 0.47 |
| Cognitive and Affective Bibliotherapy - Cognitive | Betzalel (2010) | 8 | Worry (anxiety) | RCMAS | Child/young person | Scale | Post intervention | 0-3 months | -0.11 | -0.65 | 0.42 |
| Cognitive and Affective Bibliotherapy - Cognitive | Betzalel (2010) | 8 | Anxiety total | RCMAS | Child/young person | Scale | Post intervention | 0-3 months | -0.25 | -0.79 | 0.29 |
| Cognitive and Affective Bibliotherapy - Cognitive | Betzalel (2010) | 8 | Anxiety total | RCMAS | Child/young person | Scale | Follow-up | 4-6 months | -0.46 | -1.01 | 0.08 |
| Cognitive and Affective Bibliotherapy - Cognitive | Betzalel (2010) | 8 | Physiological (anxiety) | RCMAS | Child/young person | Scale | Post intervention | 0-3 months | -0.51 | -1.06 | 0.04 |
| Cognitive and Affective Bibliotherapy - Cognitive | Betzalel (2010) | 8 | Physiological (anxiety) | RCMAS | Child/young person | Scale | Follow-up | 4-6 months | -0.52 | -1.06 | 0.03 |
| Cognitive and Affective Bibliotherapy - Cognitive | Betzalel (2010) | 8 | Worry (anxiety) | RCMAS | Child/young person | Scale | Follow-up | 4-6 months | -0.53 | -1.07 | 0.02 |
| Cognitively-Based Compassion Training (CBCT) | Reddy (2013) | 6 | Depressive symptoms (DSM IV) | QIDS-SR | Unclear | Scale | Post intervention | 0-3 months | 0.01 | -0.46 | 0.47 |
| Cognitively-Based Compassion Training (CBCT) | Reddy (2013) | 6 | Trait anxiety | STAIT/STAIS | Unclear | Scale | Post intervention | 0-3 months | -0.38 | -0.85 | 0.09 |
| Dojo: Biofeedback videogame | Schuurmans (2018) | 8 | Anxiety | SCAS | Parent/carer | Scale | Follow-up | 4-6 months | 0.02 | -0.74 | 0.78 |
| Dojo: Biofeedback videogame | Schuurmans (2018) | 8 | Anxiety | SCAS | Child/young person | Scale | Follow-up | 4-6 months | -0.12 | -0.78 | 0.53 |
| Dojo: Biofeedback videogame | Schuurmans (2018) | 8 | Anxiety | SCAS | Child/young person | Scale | Post intervention | 0-3 months | -0.14 | -0.79 | 0.52 |
| Dojo: Biofeedback videogame | Schuurmans (2018) | 8 | Anxiety | SCAS | Parent/carer | Scale | Post intervention | 0-3 months | -0.63 | -1.30 | 0.04 |
| Fostering Individualised Assistance Program (FIAP) | Clark (1994) | 72 | Anxious / depressed | CBCL | Parent/carer | Scale | 18 month follow-up | 13-18 months | 0.18 | -0.20 | 0.56 |
| Fostering Individualised Assistance Program (FIAP) | Clark (1994) | 72 | Withdrawn | CBCL | Parent/carer | Scale | 18 month follow-up | 13-18 months | -0.16 | -0.54 | 0.22 |
| Fostering Individualised Assistance Program (FIAP) | Clark (1994) | 72 | Anxious / depressed | YSR | Child/young person | Scale | 18 month follow-up | 13-18 months | -0.17 | -0.55 | 0.22 |
| Keeping foster and kinship carers supported (KEEP) | Price (2019) | 16 | Anxiety / depression | CBCL | Parent/carer | Scale | Post-test | 4-6 months | -0.15 | -0.37 | 0.08 |
| Keeping foster and kinship carers supported (KEEP) | Price (2019) | 16 | Withdrawn / depressed | CBCL | Parent/carer | Scale | Post-test | 4-6 months | -0.17 | -0.39 | 0.05 |
| Keeping foster and kinship carers supported (KEEP) | Price (2019) | 16 | Anxiety / depression - above borderline cutoff | CBCL | Parent/carer | Categorical | Post-test | 4-6 months | -0.19 | -0.48 | 0.11 |
| Keeping foster and kinship carers supported (KEEP) | Price (2019) | 16 | Withdrawn / depressed - above borderline cutoff | CBCL | Parent/carer | Categorical | Post-test | 4-6 months | -0.35 | -0.61 | -0.10 |
| Keeping foster and kinship carers supported (KEEP) | Price (2019) | 16 | Withdrawn / depressed - above clinical cutoff | CBCL | Parent/carer | Categorical | Post-test | 4-6 months | -0.43 | -0.95 | 0.09 |
| Keeping foster and kinship carers supported (KEEP) | Price (2019) | 16 | Anxiety / depression - above clinical cutoff | CBCL | Parent/carer | Categorical | Post-test | 4-6 months | -0.41 | -1.14 | 0.32 |
| Mentoring intervention for teenage pregnancy | Mezey (2015) | 52 | Anxiety / depression | GHQ | Child/young person | Categorical | 1 year | 7-12 months | *Insufficient data to calculate effect size* | | |
| Mindfulness | Jee (2015) | 10 | State anxiety | STAIT/STAIS | Child/young person | Scale | Post intervention | 0-3 months | -0.22 | -0.83 | 0.39 |
| Mindfulness | Jee (2015) | 10 | Trait anxiety | STAIT/STAIS | Child/young person | Scale | Post intervention | 0-3 months | -0.27 | -0.88 | 0.34 |
| Take Charge | Geenen (2012) | 20 | Anxiety / depression | YSR | Child/young person | Scale | 18 month follow-up | 13-18 months | -0.09 | -0.44 | 0.27 |
| Take Charge | Geenen (2012) | 20 | Anxiety / depression | CBCL | Parent/carer | Scale | 18 month follow-up | 13-18 months | -0.36 | -0.71 | 0.00 |
| Take Charge | Geenen (2012) | 20 | Withdrawn / depressed | CBCL | Parent/carer | Scale | 18 month follow-up | 13-18 months | -0.37 | -0.73 | -0.01 |
| Take Charge | Geenen (2012) | 20 | Withdrawn / depressed | CBCL | Parent/carer | Scale | 12 month follow up | 7-12 months | -0.41 | -0.77 | -0.05 |
| Take Charge | Geenen (2012) | 20 | Anxiety / depression | YSR | Child/young person | Scale | 12 month follow up | 7-12 months | -0.44 | -0.79 | -0.08 |
| Take Charge | Geenen (2012) | 20 | Anxiety / depression | CBCL | Parent/carer | Scale | 12 month follow up | 7-12 months | -0.65 | -1.02 | -0.29 |
| Triple P for Foster Parents | Job (2022) | 5 | Anxiety symptoms | PAS | Parent/carer | Scale | 6 month follow-up (m = 7 months) | 7-12 months | -0.20 | -0.68 | 0.27 |
| Triple P for Foster Parents | Job (2022) | 5 | Anxiety symptoms | PAS | Parent/carer | Scale | 12 month follow-up (m = 13 months) | 13-18 months | -0.37 | -0.84 | 0.10 |
| Wave by Wave Surf Therapy | Pereira (2020) | 21 | Anxiety | RCDAS | Child/young person | Scale | Post-test | 4-6 months | -0.05 | -0.56 | 0.46 |
| Wave by Wave Surf Therapy | Pereira (2020) | 21 | Depression | RCDAS | Child/young person | Scale | Post-test | 4-6 months | -0.07 | -0.57 | 0.44 |

#### Other mental, behavioural and neurodevelopmental disorder outcome domain

| **Intervention** | **Lead author** | **Int. length (weeks)** | **Outcome assessed** | **Tool** | **Informant** | **Data type** | **Follow-up** | **Follow-up (category)** | **Effect size** | **95% CI (low)** | **95% CI (high)** |
| --- | --- | --- | --- | --- | --- | --- | --- | --- | --- | --- | --- |
| ***Stress and post-traumatic stress outcomes*** | | | | | | | | | | | |
| Child Adult Relationship Enhancement (CARE) | Messer (2018) | 4 | PTS Arousal | TSCYC | Parent/carer | Scale | 1 month follow-up | 0-3 months | -0.72 | -1.97 | 0.53 |
| Child Adult Relationship Enhancement (CARE) | Messer (2018) | 4 | PTS Arousal | TSCYC | Parent/carer | Scale | 3 month follow-up | 4-6 months | -1.01 | -2.44 | 0.41 |
| Fostering Healthy Futures (FHF) | Taussig (2010) | 30 | Post traumatic symptoms | TSCYC | Child/young person | Scale | Post intervention | 7-12 months | -0.10 | -0.42 | 0.22 |
| Fostering Healthy Futures (FHF) | Taussig (2010) | 30 | Dissociation symptoms | TSCYC | Child/young person | Scale | Post intervention | 7-12 months | -0.13 | -0.45 | 0.19 |
| Fostering Healthy Futures (FHF) | Taussig (2010) | 30 | Post traumatic symptoms | TSCYC | Child/young person | Scale | 6 month post-intervention | 13-18 months | -0.30 | -0.63 | 0.02 |
| Fostering Healthy Futures (FHF) | Taussig (2010) | 30 | Dissociation symptoms | TSCYC | Child/young person | Scale | 6 month post-intervention | 13-18 months | -0.39 | -0.70 | -0.08 |
| Fostering Healthy Futures (FHF) | Taussig (2019) | 30 | Post traumatic symptoms | TSCYC | Child/young person | Scale | 6 month post-intervention | 13-18 months | -0.20 | -0.40 | 0.00 |
| Fostering Healthy Futures (FHF) | Taussig (2019) | 30 | Dissociation symptoms | TSCYC | Child/young person | Scale | 6 month post-intervention | 13-18 months | -0.29 | -0.49 | -0.09 |
| Life Story | Haight (2010) | 28 | PTSD / Disassociation | CBCL | Parent/carer | Scale | 12 month follow-up | 7-12 months | 0.00 | -1.01 | 1.01 |
| ***Attachment style and disorder outcomes*** | | | | | | | | | | | |
| Communication and Attachment Training for Foster Carers | Minnis (2001) | 3 | Reactive Attachment Disorder score | RADS | Parent/carer | Scale | 9 month follow-up | 7-12 months | -0.13 | -0.46 | 0.19 |
| ***Attention and hyperactivity disorder* outcomes** | | | | | | | | | | | |
| Family Minds | Adkins (2021) | 5 | Hyperactivity-inattention | SDQ | Parent/carer | Scale | Six week follow-up | 0-3 months | 0.34 | -0.21 | 0.89 |
| Fostering Changes (FC) | Moody (2020) | 12 | Hyperactivity-inattention | SDQ | Parent/carer | Scale | 3 month follow-up | 0-3 months | 0.87 | 0.59 | 1.15 |
| Fostering Changes (FC) | Moody (2020) | 12 | Hyperactivity-inattention | SDQ | Parent/carer | Scale | 12 month follow-up | 7-12 months | -0.07 | -0.35 | 0.20 |
