# Supplementary material for: Effectiveness of Mental Health and Wellbeing Interventions for Children and Young People in Foster, Kinship, and Residential Care: Systematic Review and Meta-Analysis
Source: Trauma Violence Abuse. 2024 Feb 16;25(4):2829–44. doi: 10.1177/15248380241227987 (PMC11370152; doi:10.1177/15248380241227987)
Supplement: sj-docx-6-tva-10.1177_15248380241227987 – Supplemental material for Effectiveness of Mental Health and Wellbeing Interventions for Children and Young People in Foster, Kinship, and Residential Care: Systematic Review and Meta-Analysis [file sj-docx-6-tva-10.1177_15248380241227987.docx]

**Appendix F: GRADE Assessment of Outcome Evaluations**

***Short-term Outcomes***

**Question:** Interventions for mental wellbeing compared to treatment as usual for mental health outcomes (short-term)

**Setting:** Short-term

| **Certainty assessment** | | | | | | | **Impact** | **Certainty** | **Importance** |
| --- | --- | --- | --- | --- | --- | --- | --- | --- | --- |
| **№ of studies** | **Study design** | **Risk of bias** | **Inconsistency** | **Indirectness** | **Imprecision** | **Other considerations** |  |  |  |
| **Externalising problems** | | | | | | | | | |
| 18 | randomised trials | serious^a^ | not serious | not serious | serious^b^ | none | SMD 0.30 SD lower (95% CI 0.53 to 0.08 lower) | ⨁⨁◯◯ Low |  |
| **Internalising problems** | | | | | | | | | |
| 12 | randomised trials | serious^a^ | serious^c^ | not serious | serious^b^ | none | SMD 0.35 SD lower (95% CI 0.61 to 0.08 lower) | ⨁◯◯◯ Very low |  |
| **Anxiety and depression** | | | | | | | | | |
| 8 | randomised trials | serious^a^ | not serious | not serious | serious^b^ | publication bias strongly suspected^d^ | SMD 0.26 SD lower (95% CI 0.40 to 0.13 lower) | ⨁◯◯◯ Very low |  |
| **Self-harm and suicidal ideation** | | | | | | | | | |
| 1 | randomised trials | serious^a^ | not serious | not serious | extremely serious^e^ | none | *HealthRHYTHMS (Bittman, 2009)* No effect on frequency self-harm: SMD 0.19 SD lower (95% CI 0.85 lower to 0.48 higher)  No effect on suicidal ideation: SMD 0.27 SD lower (95% CI 0.94 lower to 0.39 higher) | ⨁◯◯◯ Very low |  |
| **Subjective wellbeing** | | | | | | | | | |
| 1 | randomised trials | serious^a^ | not serious | not serious | extremely serious^e^ | none | *Wave by Wave Surf Therapy (Pereira, 2020):* No effect on health related QoL: SMD 0.25 SD higher (95% CI 0.76 higher to 0.26 lower) | ⨁◯◯◯ Very low |  |
| **Total impaired functioning** | | | | | | | | | |
| 10 | randomised trials | serious^a^ | not serious | not serious | serious^b^ | none | SMD 0.18 SD lower (95% CI 0.31 to 0.05 lower) | ⨁⨁◯◯ Low |  |
| **Total problems** | | | | | | | | | |
| 11 | randomised trials | serious^a^ | not serious | not serious | serious^b^ | none | SMD 0.15 SD lower (95% CI 0.28 to 0.02 lower) | ⨁⨁◯◯ Low |  |

**CI:** confidence interval

#### Explanations

a. All trials included appraised as 'some concerns'

b. Confidence interval includes the MID of 0.2 SD.

c. Between-study variance more than 50% of total variance in meta-analysis

d. Robust Egger's regression yields one-sided p<0.05

e. Confidence interval includes the MID on both sides of the null effect.

***Long-term Outcomes***

**Question:** Interventions for mental wellbeing compared to treatment as usual for mental health outcomes (long-term)

**Setting:** Long-term

| **Certainty assessment** | | | | | | | **Impact** | **Certainty** | **Importance** |
| --- | --- | --- | --- | --- | --- | --- | --- | --- | --- |
| **№ of studies** | **Study design** | **Risk of bias** | **Inconsistency** | **Indirectness** | **Imprecision** | **Other considerations** |  |  |  |
| **Externalising problems** | | | | | | | | | |
| 9 | randomised trials | serious^a^ | not serious | not serious | very serious^b^ | publication bias strongly suspected^c^ | SMD 0.02 SD higher (95% CI 0.17 lower to 0.20 higher) | ⨁◯◯◯ Very low |  |
| **Internalising problems** | | | | | | | | | |
| 7 | randomised trials | serious^a^ | not serious | not serious | extremely serious^d^ | none | SMD 0.03 SD lower (95% CI 0.31 lower to 0.25 higher) | ⨁◯◯◯ Very low |  |
| **Anxiety and depression** | | | | | | | | | |
| 4 | randomised trials | very serious^e^ | serious^f^ | not serious | extremely serious^g^ | none | *Fostering Individualised Assistance Program (Clark, 1994)*:  No effect on child or parent-rated anxiety/depression or withdrawal  *Triple P for Foster Parents (Job, 2022):*  No effect on anxiety symptoms  *Mentoring intervention for teenage pregnancy (Mezey, 2015)*  No effect on anxiety/depression  *Take Charge intervention (Geenen,2012)*  Lower anxiety/depression score at 12 months, self-rated (SMD 0.44 SD lower, 95% CI: 0.79 lower to 0.18 lower) and parent rated (SMD 0.65 SD lower, 95% CI: 1.02 lower to 0.29 lower)  Lower withdrawal/depression score at 12 months, parent rated (SMD 0.41 SD lower, 95% CI: 0.77 lower to 0.05 lower)  Lower anxiety/depression score at 18 months, parent rated (SMD 0.36 SD lower, 95% CI: 0.71 lower to 0.00 lower)  Lower withdrawal/depression score at 18 months, parent rated (SMD 0.37 SD lower, 95% CI: 0.73 lower to 0.01 lower) | ⨁◯◯◯ Very low |  |
| **Self-harm and suicidal ideation** | | | | | | | | | |
| 1 | randomised trials | very serious^h^ | not serious | not serious | extremely serious^d^ | none | *Mentoring intervention for teenage pregnancy (Mezey, 2015):*  No effect on reported incidents of self-harm or suicide attempts | ⨁◯◯◯ Very low |  |
| **Subjective wellbeing** SMD 0.03 SD higher (95% CI 0.63 lower to 0.69 higher) | | | | | | | | | |
| 1 | randomised trials | serious^i^ | not serious | not serious | very serious^b^ | none | *Fostering Healthy Futures (Taussig, 2010)*  Higher health related quality of life at 9 months post intervention : SMD 0.42 SD higher (95% CI 0.12 higher to 0.71 higher)  No effect on health related quality of life at 15 months: SMD 0.14 SD higher (95% CI 0.17 lower to 0.45 higher) | ⨁◯◯◯ Very low |  |
| **Total impaired functioning** | | | | | | | | | |
| 8 | randomised trials | very serious^e^ | not serious | not serious | very serious^b^ | none | SMD 0.15 SD lower (95% CI 0.40 lower to 0.09 higher) | ⨁◯◯◯ Very low |  |
| **Total problems** | | | | | | | | | |
| 6 | randomised trials | serious^j^ | not serious | not serious | extremely serious^d^ | none | SMD 0.07 SD lower (95% CI 0.38 lower to 0.25 higher) | ⨁◯◯◯ Very low |  |

**CI:** confidence interval

#### Explanations

a. All trials rated as 'some concerns' or 'high risk' but 'high risk' <25% of included trials

b. Confidence interval includes the MID of 0.2 SD and the null effect.

c. Robust Egger's regression yields one-sided p<0.05.

d. Confidence interval includes the MID on both sides of the null effect.

e. All trials rated as 'some concerns' or 'high risk' but 'high risk' >25% of included trials

f. Visual inspection of effect estimates suggests substantial spread within and between studies.

g. Evidence could not be pooled.

h. Trial rated 'high risk'

i. Trial rated as 'some concerns'

j. All trials rated as 'some concerns'
